# *Sechium edule*: Phytochemistry, Biological Activities, Potential Health Effects, Food-Industry Applications, and Future Perspectives

**DOI:** 10.3390/foods15172982

**Published:** 2026-08-25

**Authors:** Muhammad Bilal, Asad Abbas, Ralf Weiskirchen, Hammad Hafeez, Muhammad Riaz, Andleeb Zahra, Arooj Fatima, Muhammad Khurram Afzal, Hassan Raza

**Affiliations:** 1 Department of Food Science and Technology, Faculty of Food Science and Nutrition, Bahauddin Zakariya University, Multan 60800, Pakistan; sheikhbilal4666@gmail.com (M.B.); riaz@bzu.edu.pk (M.R.); andleebzahra555@gmail.com (A.Z.); aroojfatima31913@gmail.com (A.F.); 2 Department of Human Nutrition, Faculty of Food Science and Nutrition, Bahauddin Zakariya University, Multan 60800, Pakistan; khurram.afzal@bzu.edu.pk (M.K.A.); sh.raza40@yahoo.com (H.R.); 3 Institute of Molecular Pathobiochemistry, Experimental Gene Therapy and Clinical Chemistry (IFMPEGKC), RWTH University Hospital Aachen, D-52074 Aachen, Germany; 4 Department of Food Science and Technology, Faculty of Agriculture and Environment, The Islamia University of Bahawalpur, Punjab 63100, Pakistan; hammad.hafeez@iub.edu.pk

**Keywords:** *Sechium edule*, chayote, phytochemistry, cucurbitacins, antioxidant, hypoglycemic, functional food, bioactive films, metabolic syndrome, food applications

## Abstract

*Sechium edule* (Jacq.) Sw. (chayote), a neglected and underutilized Cucurbitaceae crop widely cultivated across tropical and subtropical regions, has drawn growing interest as a source of health-promoting food components. This review critically synthesizes studies published between 2000 and 2026 on its botanical features, genome characterization, nutritional value, phytochemistry, bioactivities, safety, and food-industry applications, based on literature retrieved from PubMed, Scopus, Web of Science, ScienceDirect, and the Cochrane Library. Different plant parts (fruits, leaves, seeds, tuberous roots, and peels) contain diverse bioactive compounds, including flavonoids, phenolic acids, cucurbitacins, pectin polysaccharides, and carotenoids. Reported bioactivities include antioxidant, anti-inflammatory, hypoglycemic, cardioprotective, antiproliferative, and geroprotective effects, mediated in part through Nrf2-mediated antioxidant signaling and sirtuin (SIRT1/3/5/6) upregulation. Notably, a systematic meta-analysis demonstrated a significant reduction in serum glucose (MD = −20.56; 95% CI: −29.35 to −11.77) and HbA1c following three months of chayote intake in patients with metabolic syndrome and type 2 diabetes. Industrially, chayote has been developed into fermented products, starch- and peel-derived bioactive films, ultrasound-extracted pectin, α-amylase inhibitory seed protein isolates, and probiotic encapsulation systems. Recent genomic work has further revealed a chromosome-level genome assembly, whole-genome duplication events, and a domestication history tracing to Mexico’s Oaxaca region. Collectively, this evidence positions chayote as a promising underutilized resource for food, nutraceutical, and biomedical use, while highlighting key gaps: the need for standardized clinical trials, bioavailability studies, and comprehensive safety evaluation.

## 1. Introduction

Neglected and underutilized species have gained growing interest as global food systems face nutritional insecurity, limited dietary diversity, and the increasing burden of non-communicable diseases. Among these species, *Sechium edule* (Jacq.) Sw., commonly known as vegetable pear, mirliton, choko, christophine, and chow-chow, is recognized as a versatile plant, as its fruits, leaves, tuberous roots, seeds, and shoots are all edible and nutritionally valuable [1,2]. The species originated in Mesoamerica, and recent evidence has confirmed the Oaxaca biogeographic province of southern Mexico as its center of domestication [3]. At present, chayote is grown in many tropical and subtropical regions, including parts of the Americas, Asia, Africa, and Europe [2,4].

Compared with major cucurbit crops such as bitter gourd, cucumber, melon, and watermelon, chayote remains relatively unexplored in scientific research, despite its long-standing ethnomedicinal value, widespread culinary use, and limited patent attention [1,5,6,7]. Traditional communities in Mexico, Central America, Asia, and Africa have used chayote for generations to help manage hypertension, diabetes, kidney disorders, and inflammatory conditions [8,9]. More recent pharmacological studies have supported these traditional claims, reporting antioxidant, hypoglycemic, antiproliferative, anti-inflammatory, and cardioprotective effects in different experimental models [10,11]. Importantly, recent clinical evidence from a meta-analytic study also indicates significant hypoglycemic effects in older adults with type 2 diabetes mellitus (T2DM) or metabolic syndrome (MetS) [12,13,14].

From a food science perspective, chayote has strong potential as a functional food ingredient. Its pectin has been reported to possess better rheological and antioxidant properties than commercial apple pectin [15]. In addition, its tuber starch can be modified for use in biodegradable packaging materials [16,17], its leaves provide a rich source of carotenoids and myricetin [18], and its seed protein isolates show high digestibility along with enzyme-inhibitory activity [19]. Fermentation can further enhance the flavonoid aglycone and antioxidant content of chayote-based products [20,21], suggesting its potential use in developing functional fermented foods. Recent advances in *S. edule* genomics have led to a chromosome-level genome assembly measuring 606.42 Mb and consisting of 14 chromosomes and 28,237 protein-coding genes. These studies have also identified a whole-genome duplication event approximately 25 million years ago and provided new insights into flavonoid biosynthesis, fruit development, and domestication history [22,23]. Such genomic resources help to support molecular breeding programs and improve the understanding of the genetic factors underlying phytochemical diversity in chayote [24,25].

Although evidence on *S. edule* has increased in recent years, few existing reviews integrate its genomic, phytochemical, clinical, and industrial aspects within a single synthesis. Available treatments tend to be either narrow in disciplinary scope or outdated relative to the substantial literature published subsequently. The present review addresses this gap by critically summarizing studies published between 2000 and 2026. Moreover, the present review addresses three specific gaps left unaddressed by prior syntheses: (i) integration of post-2019 genomic findings with phytochemical and bioactivity data; (ii) critical appraisal of recent meta-analytic clinical evidence and its proposed molecular mechanisms; and (iii) systematic coverage of emerging food-industry applications developed since the last comprehensive review. It also covers the botanical and genomic features of chayote, its nutritional and phytochemical composition, food-related bioactivities with evidence grading, safety profile, and major food-industry applications, including fermentation, biodegradable packaging, pectin extraction, starch modification, and protein isolation. Special attention is given to the quality of clinical evidence and the mechanisms that support its potential use in functional food development.

## 2. Search Methodology

This review identified, screened, and critically synthesized published literature on *S. edule* from PubMed, Scopus, Web of Science, ScienceDirect, the Cochrane Library, and Google Scholar, covering January 2000 to July 2026. The search strategy combined controlled vocabulary (MeSH terms, where applicable) with free-text keywords for the species and its common names (*Sechium edule*, chayote, choko, mirliton, christophine), using the Boolean operator AND to combine these with topic-specific terms (phytochemistry, nutritional composition, antioxidant, antidiabetic, anticancer, anti-inflammatory, pectin, polysaccharides, starch, fermentation, food application, bioactive film, genome, clinical trial, metabolic syndrome), and the Boolean operator OR to combine synonymous or related terms within each concept group. The exact strings applied were as follows: for PubMed, (“*Sechium edule*” (MeSH Terms) OR “*Sechium edule*” OR chayote OR choko OR mirliton OR christophine) AND (phytochemistry OR “nutritional composition” OR antioxidant OR antidiabetic OR anticancer OR “anti-inflammatory” OR pectin OR polysaccharides OR starch OR fermentation OR “bioactive film” OR genome OR “clinical trial” OR “metabolic syndrome”); for Scopus, TITLE-ABS-KEY (“*Sechium edule*” OR chayote OR choko OR mirliton OR christophine) AND TITLE-ABS-KEY (phytochemistry OR antioxidant OR antidiabetic OR anticancer OR pectin OR polysaccharides OR starch OR fermentation OR genome OR “clinical trial”); and for Web of Science, TS = (“*Sechium edule*” OR chayote OR choko OR mirliton OR christophine) AND TS = (phytochemistry OR antioxidant OR antidiabetic OR anticancer OR pectin OR starch OR fermentation OR genome OR “clinical trial”). Equivalent free-text combinations, adapted to each platform’s syntax, were applied in ScienceDirect, the Cochrane Library, and Google Scholar. After screening for relevance, full-text availability, and methodological quality, 134 peer-reviewed articles were included in the final review. Studies related to plant pathology, entomology, and food security were also considered when they contributed useful context on cultivation challenges, production limitations, or patterns of use.

## 3. Botanical, Geographical, and Genomic Characterization

*S. edule* is a perennial, monoecious climbing herb of the *Cucurbitaceae* family. It is mainly recognized by its tendrils, tuberous roots, and unusual reproductive feature: a single, endocarpic, recalcitrant seed that germinates viviparously within the fruit [26]. The plant can reach approximately 10–15 m in height and produce alternate, hastate to ovate leaves, small white campanulate flowers, and green to white pear-shaped fruits weighing about 200–500 g [1,2,20]. Considerable morphological variation has been reported among different accessions. Fruit color may range from white and light green to dark green or spiny forms, while fruit weight may vary from 60 to 560 g. Other traits, including spine density, flesh texture, and sugar content, also differ widely among landraces and cultivars [26,27].

The species is cultivated in more than 40 countries, with important production areas in Mexico, Costa Rica, Brazil, China, Japan, and India [1,2]. Recent phylogenomic analysis based on genome-wide single-nucleotide polymorphisms has identified *S. edule* as the closest wild relative of cultivated chayote. The same analysis linked its domestication to the Oaxaca biogeographic province during the Pleistocene and revealed ongoing interspecific gene flow, making species boundaries within the genus more difficult to define. Low genetic diversity and small effective population sizes in wild taxa highlight the urgent need for conservation of crop wild relatives [3]. Propagation of chayote is typically achieved through the viviparous seed or vegetative cuttings. Micropropagation protocols in RITA^®^ bioreactors have demonstrated superior plant vigor and biomass compared with semisolid medium [28]. At the same time, treatment of semi-hardwood cuttings with 1.0 g/L indole-3-butyric acid (IBA) on peat substrate achieved the highest rooting rate (94.33%), with exogenous IBA promoting nutrient metabolism, antioxidant enzyme activities, and endogenous hormone regulation [29].

The chromosome-level genome assembly of *S. edule* (606.42 Mb; 14 chromosomes; N50 scaffold = 46.56 Mb; 28,237 protein-coding genes; 65.94% repetitive sequences) was generated using Nanopore third-generation sequencing combined with Hi-C data [22]. Comparative genomics revealed that chayote and snake gourd share a common ancestor that diverged from sponge gourd, and a whole-genome duplication event occurred in chayote approximately 25 ± 4 million years ago [22]. Orphan gene analysis across eight Cucurbitaceae genomes found that chayote has the lowest orphan gene content (1.63%), suggesting high genome conservation. These orphan genes show tissue-specific expression predominantly in male flowers and may contribute to cucurbit-specific adaptive functions [30]. Genome-wide identification of GATA transcription factors in ten Cucurbitaceae species, including chayote (281 total GATA genes in 10 species; four subgroups A–D), identified stress-responsive cis-elements and segmental duplication as the main expansion mechanism [31]. Similarly, recent phylogenomic research on *S. edule* has provided important insights into its domestication history, evolutionary relationships, and genetic diversity, highlighting its significance as a neglected and underutilized crop. These findings support greater emphasis on genomic characterization, conservation of wild relatives, and sustainable utilization of *S. edule* germplasm [3]. The SSR marker landscape (363,156 SSR motifs; 42 polymorphic primer pairs; average 3.64 alleles/locus) revealed two major genetic clusters among Chinese germplasm accessions, with peel color and spine density as the most discriminating traits [24]. MADS-box gene family analysis (70 genes; 14 type I, 56 type II) has implicated specific MADS genes in male and female flower development and root tuber formation [23].

## 4. Nutritional and Phytochemical Composition

Chayote is distinguished by its low caloric density (approximately 19 kcal/100 g), very high moisture content (93–95%), and a notable folate content, which surpasses that of most common vegetables [1]. The fruit of *S. edule* contains vitamin C (7.7 mg/100 g), potassium (125 mg/100 g), calcium (17 mg/100 g), and a useful amount of dietary fiber (1.2–1.7 g/100 g) [1,32]. Compared with fruit, the shoot fractions are notably richer in ash, fiber, minerals such as calcium, potassium, magnesium, phosphorus, iron, and zinc, as well as phenolics, flavonoids, and ascorbic acid. At the same time, the leaves are particularly rich in carotenoids [32]. The seeds contain a well-balanced profile of essential amino acids (315.63 mg/g protein) and show high protein digestibility (80.3%). The tuberous roots act as an important starch reserve (15–25%), with structural characteristics similar to those of potato and cassava starch [33,34,35].

The phenolic composition of chayote has been widely investigated using HPLC-ESI-TOF-MS, UPLC-MS/MS, and related analytical platforms [18,36]. A recent metabolomic study compared domesticated and wild *S. edule* using untargeted UHPLC-ESI-QTOF-MS and targeted HPLC analyses and demonstrated substantial metabolic reprogramming associated with domestication, including differences in flavonoid, terpenoid, phenylpropanoid, lipid, nucleotide, and amino acid-related pathways [37]. The best extraction yield was obtained using 70% ethanol, producing 188 ± 2 mg phenolics/100 g dry weight. In pickled chayote during 90 days of natural fermentation, 15 phenolic compounds were quantified, and flavonoid aglycones such as luteolin, apigenin, diosmetin, and isorhamnetin increased significantly [20]. Fermentation with *Lactobacillus alimentarius* and *L. futsaii* increased apigenin derivatives, whereas *Pichia membranifaciens* supported the formation of flavonoid aglycones [20].

Chayote leaves have also shown a rich phenolic profile. UAE-HPLC analysis detected 26 phenolic compounds, with flavonols accounting for 55% of the total, mainly myricetin. Phenolic acids accounted for approximately 35%, including ferulic acid, chlorogenic acid, and (+)-catechin, while xanthophylls represented 42–85% of the total carotenoids [18]. Metabolomics and transcriptomics of chayote fruits at three storage stages identified 57 flavonoid compounds, of which 42 glycosides were significantly differentially accumulated. Exogenous phenylalanine application upregulated total flavonoid content and biosynthetic gene expression via a candidate R2R3-MYB transcription factor (FSG0057100) [36].

Among the notable enzymes identified in chayote is β-glucosidase (EC 3.2.1.21), which was purified and partially characterized as a 116 kDa protein (two 58 kDa subunits) showing homology with *Cucumis sativus* β-glucosidase. It exhibits a K_m_ of 4.88 mM, an optimal pH of 4.0, and an optimal temperature of 50 °C, and is resistant to urea and iodoacetamide [38]. A distinct isoform II of β-glucosidase forms molecular aggregates through hydrophobic amino acid interactions (39% nonpolar residues), a mechanism different from the β-glucosidase aggregating factor (BGAF) known in grasses, with a K_m_ of 4.59 mM and kcat of 10,087 µM·min^−1^ [39]. Chayote fruit also contains phenylalanine at 32.67 mg/100 g, among the lowest levels among the unconventional food plants studied, making it potentially suitable for inclusion in diets for patients with phenylketonuria to improve dietary diversity [40].

Cucurbitacins, triterpenoid compounds abundant in Cucurbitaceae, are present in bitter/wild cultivars and *Sechium* hybrids and exhibit potent antiproliferative activity [41,42,43,44]. Chayote pectin (CP) has been structurally characterized as an anionic heteropolysaccharide (CP-2) with a backbone of →4)-β-d-Galp-(1→3,6)-β-d-Galp-(1→3)-β-d-Galp-(1→4)-α-d-GalpA-(1→, with branches of α-l-Araf and β-d-Glcp [45]. Water-soluble polysaccharides (WSP) from chayote contain pectic homogalacturonan and type I rhamnogalacturonan backbones, hemicellulosic glucomannan, xyloglucan, and glucurono(arabino)xylan [46]. LC-PDA-MS analysis of different edible organs of *S. edule* identified eight flavonoids, comprising three C-glycosyl and five O-glycosyl flavones, with the highest total flavonoid content detected in the leaves (35.0 mg/10 g dry weight), followed by roots (30.5 mg/10 g) and stems (19.3 mg/10 g) [47]. The nutritional composition and bioactive compounds in different parts of *S. edule* are documented in Table 1.

## 5. Advanced Extraction Versus Solvent Extraction Methods

Emerging green extraction methods enhance the efficient isolation of phytochemicals while reducing solvent consumption, extraction time, and environmental impact [62]. Researchers have explored both traditional solvent extraction methods and novel green extraction technologies to extract bioactive phytochemicals from *S. edule*, highlighting the significant impact of the extraction method on phytochemical recovery and subsequent bioactivity. Traditional methods such as maceration, Soxhlet extraction, and reflux are widely employed because they are easy to operate and do not require expensive instrumentation. However, they yield lower phytochemical contents and require longer processing times compared with advanced methods. Comparative studies of the same plant matrix, such as chlorogenic acid extraction from chayote peel, show that UAE produced a yield of 9.34%, significantly higher than MAE (6.56%) and conventional maceration (6.88%) [63]. A parallel pattern emerged in leaf tissue, where UAE yielded 11.80 ± 1.32%, exceeding MAE (9.01 ± 1.70%) and conventional maceration (7.18 ± 1.02%) [18]. These paired comparisons were performed under controlled matrix and solvent conditions to provide direct evidence of improved mass transfer of phenolics, carotenoids, and flavonoids in cavitation-driven cell-wall disruption methods compared with conventional maceration.

In addition to yield, advanced extraction techniques are more effective for thermolabile and structurally complex bioactive compounds due to their greater selectivity. The phytochemical profile of the UAE extracts of chayote leaves showed a greater number of antioxidant compounds (myricetin, rutin, quercetin, chlorogenic acid, ferulic acid, β-carotene, xanthophylls, and α-tocopherol), resulting in stronger antioxidant activity, particularly in the ABTS and FRAP assays, than MAE or maceration [18]. In addition, ultrasound-assisted alkaline extraction of chayote seed protein yielded 88%, compared with the conventional alkaline extraction method (58%) and ultrasound bath method (72.4%), with minimal thermal effects that helped prevent protein denaturation. The resulting isolate showed the highest purity (66.7%), the highest phenolic content (7.22 mg GAE/g DW), and strong α-amylase inhibition (74%) [19]. Similarly, ultrasound-assisted extraction (UAE) has been shown to recover cell-wall polysaccharides effectively from chayote fruit. Two polysaccharide fractions, CP-1 (41.1 kDa) and CP-2 (15.6 kDa) were obtained after ultrasonic treatment (180 W, 70 °C, 40 min) and purification by chromatography [45]. Conventional heating extraction has also been used to recover and characterize non-starch polysaccharides (NSP) from chayote [64].

Conventional solvent-based methods, on the other hand, remain useful when polarity-based fractionation is the primary goal, rather than raw yield. Sequential and reflux extraction using solvents of increasing polarity has enabled systematic mapping of phytochemical distribution in chayote, with the ethyl acetate fractions performing best. They exhibited the highest phenolic content in fruit (3.21 g GAE/100 g extract), the highest flavonoid and carotenoid content in leaves (11.64 g QE/100 g and 12.73 g β-carotene equivalents/100 g, respectively) and the most potent radical-scavenging activity in pedicel tissue (DPPH IC_50_ = 1.3 µg/mL) [65]. Polarity-based fractionation of chayote pulp and leaves using methanol and ethyl acetate showed a strong correlation (r = 0.99) between TPC (mM g^−1^ extract) and antioxidant capacity (mM TE g^−1^ extract), with the ethyl acetate fraction showing an antioxidant capacity of 951.73 ± 29.14 mM TE g^−1^ extract [66]. Hydroalcoholic maceration (ethanol:water 60:40 or 70:30) of chayote tuberous roots, on the other hand, has been widely employed to obtain cinnamic and coumaric acid derivatives, which have been linked to vasorelaxant and antihypertensive activity, with reproducible extraction yields of approximately 8.9% in independent studies [53,67,68]. The results showed that conventional maceration is also methodologically appropriate for targeted fractionation and bioassay-guided isolation strategies, where compound-class selectivity is governed by solvent polarity rather than extraction intensity.

Another difference between the two extraction paradigms is compound stability and the retention of downstream bioactivity. Advanced techniques such as UAE, which use short extraction times and can operate at lower or controlled temperatures, appear to retain heat-labile antioxidants and structurally complex glycosides better, as evidenced by the higher recovery of carotenoids and tocopherols using UAE compared with conventional methods for both peel [63] and leaf matrices [18]. By contrast, conventional extraction and roasting or steaming processes have been shown to decrease phenolic content and related bioactivity in extracts of chayote peel, chayote leaf, and chayote pulp, indicating that these compound classes are sensitive to prolonged thermal exposure, irrespective of the extraction technique [51]. However, some traditional methods have particular value for selectively extracting specific non-phenolic bioactive classes: Soxhlet defatting followed by ethanolic maceration yielded not only alkaloids and saponins but also cardenolides and bufadienolides, which are not targeted by ultrasound-optimized protocols [69]. Sequential hexane-to-methanol extraction of whole fruit allowed chromatographic isolation of the cucurbitacin fraction, which showed antiproliferative activity against breast cancer cell models [70]. Further methanolic fractionation isolated cucurbitacins B, D, E, and I, as well as a panel of flavonoids that exhibited activity against HeLa cells [43]. These findings suggest that the choice of extraction method for *S. edule* should not rely solely on maximizing yield but should also consider the target compound class.

Evidence from various studies on *S. edule* suggests that advanced and conventional extraction methods have complementary rather than mutually exclusive functions. UAE proved more effective than MAE and conventional maceration in recovering bioactive compounds such as phenolics from chayote peel and leaves [18,63]. Similarly, UAE produced higher extraction yields and better protein functional properties from chayote seeds than conventional alkaline extraction [19]. Furthermore, UAE offers additional advantages for retaining antioxidant properties and the structural stability of recovered polysaccharides [45,64]. For isolating specific classes of compounds, such as cucurbitacins, alkaloids, and saponins, conventional methods like reflux, Soxhlet extraction, or sequential solvent maceration [43,53,65,66,67,69,70] are better known and established. Comprehensive reporting of yield and composition in future studies using response-surface-optimized UAE or MAE protocols, in combination with conventional protocols, would greatly facilitate comparative conclusions and enable more rational selection of extraction methods by compound class for valorizing *S. edule*. Table 2 summarizes advanced extraction strategies compared with traditional methods, along with their phytochemical profiles and bioactive potential.

## 6. Biological Activities and Potential Health Effects

### 6.1. Antioxidant Activity

Oxidative stress, defined as an imbalance between ROS production and endogenous antioxidant defenses, is a crucial factor in the pathogenesis of type 2 diabetes mellitus (T2DM), cardiovascular disorders, neurodegenerative diseases, and physiological decline during aging [76]. Fruits and vegetables are therefore a natural source of dietary antioxidants with the potential to alleviate oxidative damage via redox-sensitive signaling and have been the subject of much interest [77,78,79]. *S. edule* has been identified as a food of interest because of its high content of phenolic compounds, flavonoids, cucurbitacins, carotenoids, and vitamin C, which contribute to its antioxidant properties [18,32].

The antioxidant activity of various parts of *S. edule* (leaves, young shoots, and fruits) has been found to be significant in various standard antioxidant assay systems. The leaves have the highest activity in all three assays (DPPH, ABTS, FRAP) compared with the fruits and stems, which is attributed to the high TPC and flavonoid contents [18,32]. Methanolic and hydroalcoholic extracts have been subjected to bioactivity-guided phytochemical investigations, which revealed chlorogenic acid, rutin, quercetin, naringenin, myricetin, galangin, and cucurbitacins as the most significant phytochemicals responsible for this free-radical scavenging property and associated reduction in oxidative stress [80]. In a phytopathogen-screening study relevant to chayote, *Chryseobacterium* sp., a bacterium associated with necrotic leaf symptoms in *S. edule*, was included among the test organisms. Phenolic compounds identified from selected Mexican cloud-forest plants, including vanillin, trans-cinnamic acid, scopoletin, and umbelliferone, exhibited bacteriostatic activity against *Chryseobacterium* sp. [81].

These antioxidant effects are chemically defined and have measurable biological effects in cell and animal models. Shoot extracts of *S. edule* were shown to be potent in reducing intracellular oxidative stress and activating AMP-activated protein kinase (AMPK), which is associated with enhanced lipid metabolism and metabolic homeostasis [82]. In mice challenged with benzo[a]pyrene-induced micronuclei, unfiltered fresh chayote juice provided around 70% genoprotection, which was thought to be due to the synergistic effect of dietary fiber and polyphenolic constituents [83]. Similarly, a hydroalcoholic extract of *S. edule* roots was able to prevent the oxidative stress induced by angiotensin II in endothelial tissue by maintaining the bioavailability of nitric oxide, reducing the generation of ROS in the endothelium, and improving endothelial function [84] in a physiologically relevant context. Preclinical evidence has been supported by clinical studies that demonstrate that antioxidant properties are also seen in humans. *S. edule* var. *nigrum spinosum* (500 mg, thrice a day for 6 months) significantly elevated total antioxidant status (TAS) and the activity of superoxide dismutase (SOD), catalase (CAT) and glutathione peroxidase (GPx) in older adults with metabolic syndrome while reducing total oxidant status (TOS) and oxidative stress index, and upregulating Nrf2 mRNA expression by approximately 47% [85].

The antioxidant capacity is also influenced by processing methods, in addition to the raw plant material. The fermented pickled chayote showed significantly higher TPC, TFC, and DPPH/ABTS radical-scavenging activity than the unfermented material, and extracts of these fermented products did not induce oxidative injury in HepG2 cells challenged with H_2_O_2_, indicating reduced ROS accumulation and strengthened endogenous antioxidant capacity after fermentation [22]. The same classes of phytochemicals found in the initial chemical assays were found in Sechium hybrids and exhibited similar antioxidant and anti-inflammatory properties, including the ability to reduce lipid peroxidation, increase GPx activity, suppress TNF-α production, enhance IL-10 levels, and protect against hypochlorous acid–induced membrane damage [11]. *S. edule* contains high levels of antioxidants, such as phenolics, flavonoids, and vitamin C, which help to reduce oxidative stress associated with metabolic syndrome, diabetes, and cardiovascular disease. These benefits have been supported by clinical and animal studies, which demonstrate an improvement in antioxidant markers, decreases in oxidative damage, and improvements in endothelial function with chayote supplementation. Its antioxidant activity is further enhanced by processing such as fermentation, during which its antioxidant compounds such as chlorogenic acid and quercetin contribute to its antioxidant and antimicrobial activity.

### 6.2. Antidiabetic Activity

The antidiabetic activity of *S. edule* is supported by clinical trial data, as well as by a convergent body of pharmacological, experimental, and mechanistic evidence. A systematic review and meta-analysis of three clinical trials demonstrated that chayote supplementation significantly reduced fasting serum glucose in older adults with T2DM or metabolic syndrome. The pooled mean difference was −20.56 mg/dL (95% CI: −29.35 to −11.77; *p* < 0.0001) after 3 months and −12.96 mg/dL (*p* = 0.004) after 6 months of supplementation, with corresponding HbA1c reductions of 1.12% and 0.92% at the same timepoints [12]. In a separate clinical investigation, 6 months of chayote supplementation in older adults with T2DM significantly increased mRNA expression of sirtuin genes (SIRT1 +52%, SIRT3 +69%, SIRT5 +62%, SIRT6 +69%) and improved redox status, reducing total oxidant status by 50% while increasing total antioxidant status by 44% [84]. Critically, both trials draw from an overlapping, relatively narrow demographic in older adults with T2DM. The sirtuin and redox findings are clinically observed but mechanistically unexplained at the clinical level, since the trials measured these biomarkers without establishing causality to the glucose-lowering effect, a gap that the animal and mechanistic evidence below only partially addresses.

Preclinical evidence from diabetic animal models offers biological plausibility for the clinical signal, though the translational bridge between these models and the human trials above remains indirect. In db/db mice, 8 weeks of chayote pectin treatment increased GLP-1 secretion and hepatic glycogen storage, and decreased HOMA-IR, alongside favorable shifts in gut microbiota composition by enhancing *Ruminococcaceae*, *Muribaculaceae*, *Alloprevotella*, *Rikenella*, *Parabacteroides*, and decreasing the *Firmicutes/Bacteroidetes* ratio [10]. In a streptozotocin-induced diabetic mouse model, methanolic H387-07 fruit extracts lowered glycated hemoglobin and fasting glucose, improved lipid metabolism, and protected liver, kidney, and pancreatic tissue from diabetes-associated histopathological damage [86]. In a rat model, pretreatment with fresh chayote juice significantly mitigated starch-induced postprandial hyperglycemic excursion. Although the juice exhibited antioxidant activity, the antihyperglycemic effect was not attributable to its polyphenol content, suggesting a possible contribution from proteins and other macro- or micronutrients [87]. A critical limitation worth noting is that these three animal studies used three different preparations: pectin extract, methanolic fruit extract, and fresh juice, each with distinct phytochemical profiles and likely different active constituents. This heterogeneity means that animal evidence, while directionally consistent with the clinical trials, cannot be treated as a single validated intervention; instead, it suggests that multiple, potentially independent bioactive fractions of chayote may each contribute to glycemic effects, with direct implications for future standardization efforts.

In vitro enzymatic assays provide evidence for a potential carbohydrate-digestion-related mechanism, as chayote peel extract inhibited α-amylase activity with an IC_50_ of 0.2 mg/mL [50]. This in vitro finding is directionally consistent with the significant reduction in starch-induced postprandial glycemic excursion observed in rats pretreated with fresh chayote juice [87]; however, because the studies used different experimental systems, the mechanistic connection remains inferential. Collectively, the clinical, animal, and in vitro evidence points to several complementary mechanisms, including enhanced incretin and insulin-sensitizing signaling through increased GLP-1 secretion, PI3K/Akt activation, and AMPK activation [10]; digestive enzyme inhibition through α-amylase inhibition by chayote peel extract [50]; gut microbiota modulation through favorable bacterial population shifts [10]; and antioxidant and redox regulation through sirtuin gene upregulation and improved oxidant/antioxidant status [84].

A major shortcoming of the existing mechanistic evidence is the variety of chayote preparations and experimental systems used, which limits the ability to directly compare results across studies. In db/db mice, chayote pectin corrected multiple interconnected pathways in the gut–liver axis, such as gut microbiota/SCFAs/GLP-1, PI3K/AKT/FoxO1, and GPR43/AMPK/FoxO1 signaling [10]. On the other hand, chayote peel extract showed α-amylase inhibition in vitro [50], while fresh chayote juice was non-inhibitory against the main carbohydrate-digesting enzymes tested, including amylase, α-amylase, and amylase, in reducing the starch-induced postprandial glycemia [87,88]. In older adults with type 2 DM, supplementation with powdered *S. edule* var. *nigrum spinosum* (1.5 g/day) was correlated with changes in sirtuin gene expression and redox-related parameters [84]. Therefore, direct mechanistic comparison between preparations and experimental models is not yet possible. This means that the mechanistic picture is a composite inference assembled across incompatible experimental systems rather than a validated, unified pathway, a distinction that should be made explicit since it directly affects how much mechanistic certainty the current evidence actually supports. The clinical sirtuin/redox findings in particular remain mechanistically undercharacterized, as no animal or in vitro study in this evidence base has yet demonstrated that chayote-induced sirtuin upregulation causally drives the glucose-lowering effect rather than being a parallel, correlated response to supplementation [84]. Overall, the directional consistency across all three evidence tiers clinical glucose and HbA1c reduction, animal-model glucose and hepatic improvement, and in vitro enzyme inhibition provides reasonable biological plausibility for chayote’s antidiabetic activity as a whole-food or extract-based intervention. However, the current evidence base cannot yet specify which chayote fraction, whether pectin, polyphenol-rich juice, or whole fruit extract, is responsible for which clinically observed effect, and this gap should be prioritized in future mechanistic and standardization studies alongside the larger, multicenter clinical trials needed to confirm long-term efficacy and establish robust dosage recommendations.

### 6.3. Anti-Inflammatory Activity

Chronic low-grade inflammation is an important pathological hallmark of MetS, obesity, atherosclerosis, hypertension, and type 2 diabetes mellitus. Activation of inflammatory signaling pathways leads to chronic endothelial dysfunction, oxidative stress, lipid accumulation, and vascular remodeling, which in turn increase cardiovascular risk [89,90]. As a result, a range of dietary strategies that can simultaneously influence inflammatory mediators and oxidative stress has gained significant interest. Among these, *S. edule* has shown promising anti-inflammatory and cardioprotective effects in experimental and clinical studies. Isolation of water-soluble polysaccharides from *S. edule* showed the ability to inhibit macrophage-mediated inflammatory responses, highlighting their relevance in atherosclerosis. In THP-1 macrophages activated with cholesterol crystals, WSP potently inhibited IL-1β and pro-inflammatory chemokine secretion, reduced intracellular lipid accumulation, increased the expression of liver X receptor-α (LXRα), suppressed matrix metalloproteinase-9 (MMP-9), inhibited the NLRP3 inflammasome priming, and reduced caspase-1 activation [46]. Based on these results, chayote polysaccharides could help to delay the development of atherosclerosis by positively influencing cholesterol efflux and also preventing inflammasome-driven inflammation.

These anti-inflammatory activities are also corroborated by clinical investigations. Among older adults with MetS, 6-month supplementation with *S. edule* significantly lowered systolic blood pressure, diastolic blood pressure, HbA1c, TBARS, 8-isoprostanes, 8-hydroxy-2′-deoxyguanosine (8-OHdG) and oxidative stress score, and increased HDL-cholesterol, total antioxidant status (TAS), superoxide dismutase (SOD), and the anti-inflammatory cytokine IL-10. These biochemical improvements were observed along with a reduced number of MetS diagnostic criteria, suggesting an overall improvement in cardiometabolic health [13]. Other exploratory clinical studies also showed that long-term chayote supplementation led to a significant decrease in circulating TNF-α, which further confirms its systemic anti-inflammatory effects [76].

Experimental studies have shown complementary molecular mechanisms underlying these observations. Notably, extracts from Sechium hybrids inhibited TNF-α production and enhanced glutathione peroxidase and IL-10 activity in mice, suggesting modulation of inflammatory and antioxidant pathways [11]. The results indicate that phenolic compounds, flavonoids, and cucurbitacins act synergistically in regulating the inflammatory cytokine balance. Experimental hypertension models have also provided evidence of cardiovascular protection. Lombardo-Earl et al. found that hydroalcoholic root extracts of *S. edule* effectively reduced mean arterial pressure and induced significant vasorelaxation in angiotensin II-induced hypertensive rats and rat aortic rings [53]. Bioactivity-guided fractionation indicated that the antihypertensive activity was largely due to cinnamic acid derivatives and related phenolic compounds. Similarly, Trejo-Moreno et al. showed that an acetone fraction of *S. edule* roots was beneficial in improving endothelial function in mice with vascular dysfunction induced by angiotensin II [91]. The extract inhibited oxidative stress in vascular tissue and enhanced endothelial responsiveness, suggesting that it could protect against endothelial injury caused by hypertension and cardiovascular diseases. These observations have been extended recently to hepatic and metabolic inflammation. Alvarado-Ojeda et al. [67] showed that the hydroalcoholic root extract significantly decreased hepatic concentrations of the cytokine tumor necrosis factor alpha (TNF-α), interleukin 1β (IL-1β), interleukin 6 (IL-6), and transforming growth factor-β (TGF-β) in a murine model of non-alcoholic steatohepatitis (NASH) induced by angiotensin II, and also decreased hepatic concentrations of triglycerides and hepatic fibrosis. The results indicate that the anti-inflammatory effect of *S. edule* is not limited to vascular tissues but also extends to metabolic tissues closely related to cardiometabolic disease.

Overall, *S. edule* exhibits marked anti-inflammatory and cardioprotective properties, and the polysaccharides have been found to inhibit macrophage-mediated inflammation and lower the levels of markers associated with atherosclerosis formation. In clinical trials, chayote extracts reduced blood pressure, oxidative stress markers, and tumor necrosis factor (TNF-α) and increased anti-inflammatory cytokines such as interleukin-10 (IL-10) in older adults with metabolic syndrome. Animal studies also support these benefits, indicating that chayote extracts have a wide range of cardiometabolic effects, including lowering inflammatory cytokines in both vascular and liver tissue, improving endothelial function, and reducing hypertension.

### 6.4. Antiproliferative and Anticancer Activity

Although significant progress has been achieved in chemotherapy, radiotherapy, targeted therapy, and immunotherapy, the effectiveness of these treatments is often limited by systemic toxicity, multidrug resistance, tumor recurrence, and lack of selectivity for malignant cells. As a result, interest has grown in identifying plant-derived bioactive compounds that can simultaneously modulate several oncogenic pathways while exhibiting reduced toxicity toward normal tissues. *S. edule* is an edible plant under preclinical investigation for anticancer phytochemical discovery, with cucurbitacins and polyphenolic compounds demonstrating notable antiproliferative activity in experimental cancer models. Preclinical studies to date have evaluated the bioactive compounds of *S. edule* against a range of cancer cell lines, including cervical carcinoma, breast cancer, leukemia, and fibrosarcoma models, where they have been shown to modulate apoptosis, cell-cycle regulation, oxidative stress, and proliferative signaling pathways [92,93]. It should be noted, however, that this evidence base is derived almost entirely from in vitro cell-line studies and a limited number of animal experiments; no controlled clinical trials have evaluated the anticancer efficacy of *S. edule* in humans, and these findings should therefore be interpreted as preliminary and hypothesis-generating rather than indicative of therapeutic readiness.

The antiproliferative activity of *S. edule* extracts observed in vitro appears to be genotype-dependent and influenced by phytochemical composition, extraction solvent, and the concentrations of cucurbitacins and flavonoids. In these experimental models, wild and hybrid genotypes have consistently shown significantly higher in vitro activity than commercial cultivars, highlighting the importance of germplasm selection as a valuable resource for future pharmacological investigations and functional food development [42,43,44,92,93]. This variability is attributed to differences in secondary metabolite profiles, including cucurbitacins B, D, E, and I, as well as flavonoids such as rutin, quercetin, myricetin, naringenin, phloretin, apigenin, and galangin [41,42]. Bioactivity-guided fractionation further showed that enriched chromatographic fractions had significantly higher antiproliferative activity than crude extracts [39,74,94], highlighting that purification of potent phytochemicals can enhance biological activity under experimental conditions.

Cervical carcinoma cell lines have been the focus of several in vitro studies. The Perla Negra cultivar, developed from a cross between *S. edule* var. *nigrum minor* and *S. edule* var. *amarus sylvestris*, exhibited pronounced cytotoxic activity against HeLa cells in culture (IC_50_ = 1.85 µg/mL). While showing substantially lower toxicity toward normal human lymphocytes in vitro (IC_50_ ≈ 30 µg/mL), indicating a favorable therapeutic selectivity index [42]. Further studies using chromatographic separation showed that in vitro antiproliferative activity was concentrated in specific bioactive fractions rather than distributed across all fractions [94], suggesting that enrichment of active components could be a useful strategy for future preclinical optimization.

Breast cancer cell lines have also been examined as an in vitro target for *S. edule* phytochemicals. Dichloromethane fruit extracts exhibited high potency against MCF-7 cells in cell cultures, with an IC_50_ of 0.58 µg/mL, and measurable antiproliferative activity was observed at concentrations as low as 0.07 µg/mL under the same in vitro conditions used for doxorubicin comparison [70]. Further studies showed that methanolic extracts and chromatographic fractions with high methanolic content effectively inhibited proliferation of both triple-negative MDA-MB-231 and estrogen receptor-positive MCF-7 breast cancer cell lines in vitro, suggesting that the antiproliferative effect is not restricted to estrogen receptor phenotypes alone [41]. This inhibition across distinct breast cancer cell line phenotypes suggests that the in vitro antiproliferative activity may be mediated by multiple cellular targets rather than a single common pathway.

Methanolic extracts of the H-387-07-GISeM genotype showed a significant inhibitory effect against the proliferation of leukemia cell lines such as P388, J774, and WEHI-3, with IC_50_ values below 1.3 µg/mL in vitro [93]. Notably, these extracts showed minimal cytotoxicity to normal bone marrow mononuclear cells in the same experimental system, suggesting selective activity toward malignant cells. Morphological and biochemical analyses identified classical features of apoptotic cell death, including apoptotic body formation, phosphatidylserine externalization, and DNA fragmentation [93]. The observed cytotoxicity involves activation of programmed cell death pathways rather than purely nonspecific toxicity, though this interpretation remains based on in vitro observations alone. Reported molecular events include phosphatidylserine externalization, DNA fragmentation, formation of apoptotic bodies, mitochondrial dysfunction, cytochrome c release, and activation of caspase-3, consistent with engagement of the intrinsic mitochondrial apoptosis pathway [92]. Little or no involvement of the extrinsic caspase-8 pathway has been reported in cervical cancer cell models, suggesting that the intrinsic apoptotic pathway may predominate in these particular in vitro systems [92]. Several studies reported that apoptosis was accompanied by dose-dependent reductions in cell viability broadly consistent with the known biological activities of cucurbitacins, as multitarget triterpenoids capable of promoting apoptosis and modulating cell-cycle progression, cytoskeletal organization, and oncogenic signaling in various cancer cell models.

**Figure 1:**
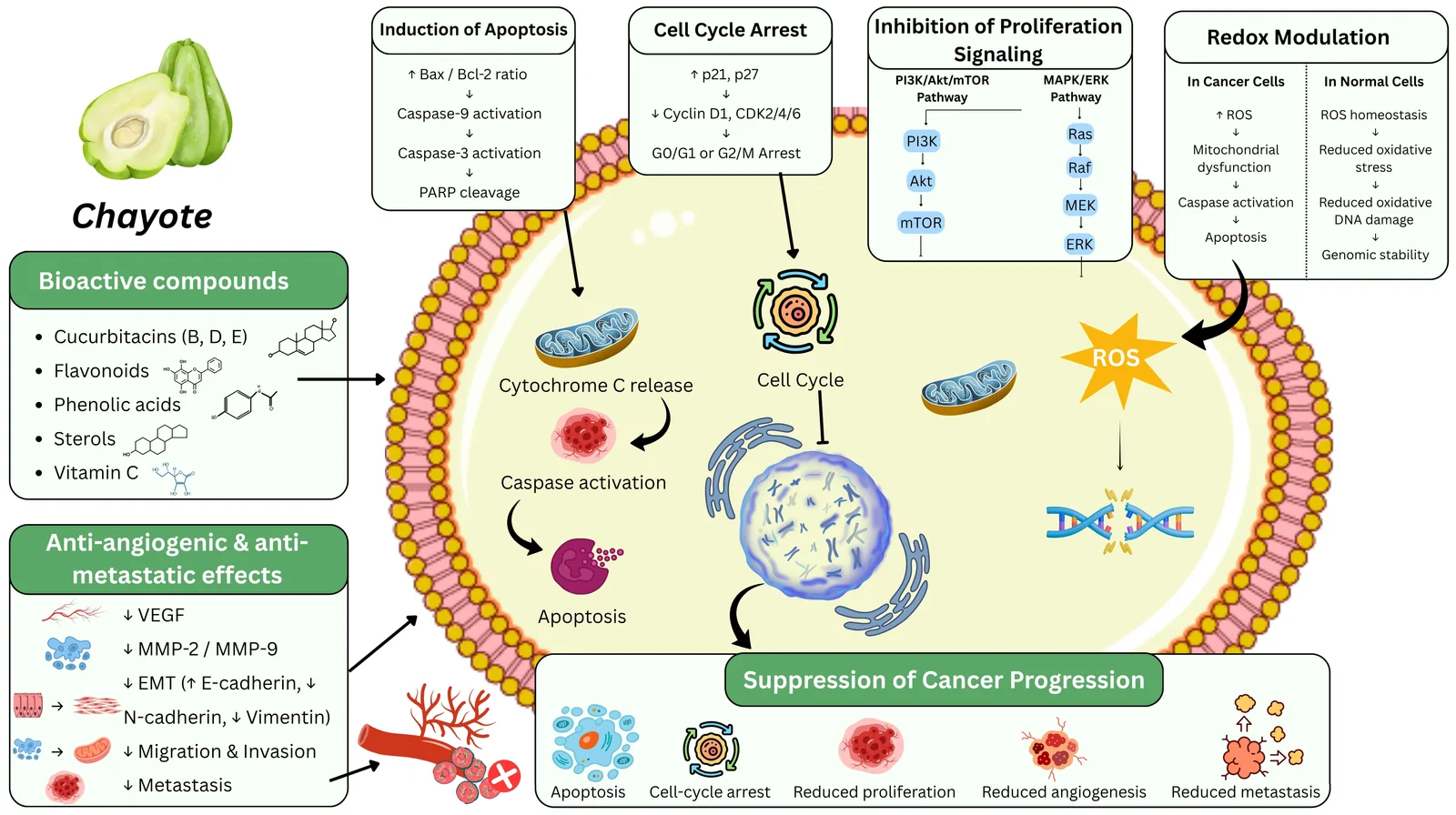
Molecular mechanisms underlying the anticancer bioactivity of *S. edule* (chayote) extracts and their major bioactive compounds. Chayote-derived bioactive compounds, including cucurbitacins (B, D, E), flavonoids, phenolic acids, sterols, and vitamin C, exert multitargeted anticancer effects through four interconnected mechanistic axes. (1) Induction of apoptosis: an increased Bax/Bcl-2 ratio triggers mitochondrial cytochrome c release, sequential activation of caspase-9 and caspase-3, and cleavage of poly(ADP-ribose) polymerase (PARP), culminating in programmed cell death. (2) Cell-cycle arrest: upregulation of the cyclin-dependent kinase inhibitors p21 and p27 suppresses cyclin D1 and cyclin-dependent kinases 2, 4, and 6 (CDK2/4/6), halting cell cycle progression at the G_0_/G_1_ or G_2_/M checkpoints. (3) Inhibition of proliferative signaling: downregulation of the phosphoinositide 3-kinase/protein kinase B/mechanistic target of rapamycin (PI3K/Akt/mTOR) pathway and the rat sarcoma (Ras)/rapidly accelerated fibrosarcoma (Raf)/mitogen-activated protein kinase (MEK)/extracellular signal-regulated kinase (ERK) pathway, i.e., the MAPK/ERK pathway, together suppress cancer cell proliferation. (4) Redox modulation: chayote extracts selectively elevate reactive oxygen species (ROS) in cancer cells, driving mitochondrial dysfunction, DNA damage, and caspase-mediated apoptosis, while preserving ROS homeostasis and genomic stability in normal cells, indicating selective cytotoxicity. Downstream of these mechanisms, chayote extracts additionally exert anti-angiogenic and anti-metastatic effects via downregulation of vascular endothelial growth factor (VEGF) and matrix metalloproteinases 2 and 9 (MMP-2/MMP-9), and by reversing the epithelial–mesenchymal transition (EMT) program (increased E-cadherin; decreased N-cadherin and vimentin), collectively reducing cancer cell migration, invasion, and metastasis. Together, these convergent pathways suppress cancer progression through apoptosis, cell-cycle arrest, reduced proliferation, reduced angiogenesis, and reduced metastasis [41,42,43,44,70,92,93,94,95].

Preliminary toxicological and pharmacokinetic data provide some early support for further investigation, though these remain limited in scope. IN an acute toxicity study of methanolic fruit extract of *S. edule* var. *nigrum spinosum*, an LD_50_ > 5000 mg/kg was observed in CD-1 mice, and no obvious acute effects or marked changes in blood chemistry or blood cell counts were observed at the doses tested [44]. Similarly, the selective cytotoxicity of *S. edule* phytochemicals observed toward malignant cells, relative to minimal effects on normal lymphocytes and bone marrow mononuclear cells in vitro [43], is a preliminary but encouraging observation that would need confirmation in more comprehensive in vivo toxicology and efficacy studies before any translational claims can be made. Pharmacokinetic data remain similarly preliminary: following oral administration of H387-07 fruit extract (125 mg/kg) in an animal model, cucurbitacin B was rapidly absorbed and distributed into systemic circulation, reaching a C_max_ of approximately 37.56 µg/mL at 1 h. Cucurbitacin IIA (CIIA) was also identified in plasma for the first time, indicating that biologically active cucurbitacins can achieve systemic bioavailability after oral administration in this animal model [41], a finding that, while promising for future pharmacokinetic characterization, does not by itself establish anticancer efficacy or safety in vivo, let alone in humans. Overall, the cumulative evidence to date supports *S. edule* phytochemicals as having demonstrable preclinical antiproliferative and cytotoxic activity across several in vitro cancer models, with limited early-stage pharmacokinetic and acute toxicity data from animal studies. This body of work justifies continued preclinical investigation, including more extensive in vivo efficacy and toxicology studies, but does not yet constitute evidence supporting *S. edule* as a candidate for cancer treatment in humans, and this distinction should be maintained throughout the manuscript’s framing of these findings. Figure 1 shows the anticancer mechanisms of different bioactive compounds present in chayote.

Overall, the data in Table 3 indicate that *S. edule* exhibits broad-spectrum antiproliferative activity through selective cytotoxicity, mitochondrial apoptosis, DNA fragmentation, phosphatidylserine externalization, cytochrome c release, caspase-3 activation, inhibition of cancer cell proliferation, and enrichment of cucurbitacin-rich bioactive fractions. However, despite these promising results, available research remains largely restricted to in vitro studies, and only a few studies have evaluated efficacy in animal tumor models; no controlled clinical trials have been performed to determine anticancer effects in humans. Future research should therefore prioritize standardized extract preparation, comprehensive phytochemical characterization, pharmacokinetic and pharmacodynamic evaluation, mechanistic validation in orthotopic and xenograft tumor models, and ultimately, well-designed clinical studies before any therapeutic role of *S. edule* in cancer prevention and treatment can be established.

### 6.5. Geroprotective and Antiaging Effects

As people age, the body experiences progressive oxidative stress, chronic low-grade inflammation, mitochondrial dysfunction, genomic instability, and telomere attrition, all of which contribute to frailty, metabolic disorders, cardiovascular disease, and functional decline. There is thus great interest in functional foods that can regulate the molecular mechanisms involved in healthy aging. Few edible plant species have shown measurable effects on biomarkers of biological aging in human intervention studies, and *S. edule* is one of the few for which such effects have been reported. The most striking clinical finding is that telomere shortening is reduced in older adults with MetS following supplementation with *S. edule* (1.5 g per day for 6 months). The participants who were given chayote had significantly less telomeric DNA loss and significantly reduced levels of certain oxidative damage markers such as lipoperoxides, protein carbonyls, 8-hydroxy-2′-deoxyguanosine (8-OHdG), and total oxidant status (TOS), as well as significantly elevated levels of total antioxidant status (TAS) compared with the placebo group [100]. Preservation of telomere integrity may be linked to enhanced redox homeostasis rather than direct stimulation of telomere elongation, given that oxidative DNA damage is one of the main factors responsible for telomere erosion.

This interpretation is also supported by subsequent clinical studies showing that serum telomerase levels remained unaffected and did not significantly increase after three months of *S. edule* supplementation [8]. It may be that maintaining telomerase activity stabilizes normal function rather than promoting uncontrolled activation of proliferative pathways. This is especially significant, since increased telomerase activity has been linked to malignant transformation, and maintaining telomere integrity by reducing oxidative stress is a physiologically desirable pathway for healthy aging. Further mechanistic support comes from sirtuin expression studies. In older adults with T2DM, six months of chayote supplementation increased the mRNA expression of SIRT1, SIRT3, SIRT5, and SIRT6, reduced systemic oxidative stress, and enhanced antioxidant capacity. These proteins play a central role in regulating longevity pathways and coordinate mitochondrial bioenergetics, DNA repair, genomic stability, cellular metabolism, and adaptive responses to oxidative stress. In particular, SIRT1 and SIRT6 are involved in chromatin remodeling and maintenance of genome integrity; SIRT3 and SIRT5 control the activity of mitochondrial enzymes and the detoxification of reactive oxygen species [84].

Geroprotective properties of *S. edule* also align with its previously reported antioxidant and anti-inflammatory activities. Clinical studies have shown that antioxidant enzymes such as superoxide dismutase, catalase, and glutathione peroxidase increase, while lipid peroxidation and oxidative DNA damage decrease [8,91,100]. Furthermore, activation of the Nrf2 antioxidant defense pathway after *S. edule* supplementation [83] may offer another molecular mechanism by which cellular homeostasis is maintained during aging, as Nrf2 regulates the transcription of many cytoprotective and antioxidant genes that together help prevent oxidative damage associated with aging. Collectively, the available evidence indicates that the geroprotective activity of *S. edule* is mediated through multiple complementary mechanisms, including reduction in oxidative DNA damage, attenuation of lipid and protein oxidation, enhancement of endogenous antioxidant defenses, activation of the Nrf2 signaling pathway, upregulation of SIRT1, SIRT3, SIRT5, and SIRT6 expression, maintenance of telomerase homeostasis, and preservation of telomere integrity [91,100]. Rather than targeting a single pathway in aging, chayote appears to affect multiple interconnected pathways related to cellular resilience and metabolism. However, the currently available evidence comes mainly from a small number of clinical trials involving elderly individuals with metabolic conditions. Further studies in healthy aging populations, with longer intervention periods and other aging-related outcomes such as mitochondrial function, cellular senescence, epigenetic regulation, proteostasis, and immune aging, should be conducted to shed more light on the role of *S. edule* as a functional food for healthy longevity.

*S. edule* exhibits significant geroprotective properties. In clinical trials, supplementation with *S. edule* was associated with decreased telomere shortening and oxidative DNA damage in elderly people with MetS, not necessarily through direct stimulation of telomerase activity but rather through improved redox balance. Additionally, supplementation increases the expression of important longevity-related genes in the sirtuin family, including SIRT1, SIRT3, SIRT5, and SIRT6, which are responsible for mitochondrial function, DNA repair, genomic stability, and reduced systemic oxidative stress. The anti-aging effects align with chayote’s antioxidant activity and include activation of the Nrf2 pathway and antioxidant enzyme activity, but these effects have been reported only in small clinical trials in elderly people with metabolic diseases.

## 7. Applications in Food Systems

*S. edule* is used in a variety of applications in food systems, including fermentation, which increases phenolic content, bioavailability, and the production of functional metabolites such as folate; pectin extraction, which produces a valuable ingredient rich in phenolics and antioxidant compounds with prebiotic and antidiabetic properties; and starch and by-product valorization for biodegradable packaging films with antimicrobial and antioxidant properties. Its seeds, shoots, and leaves are being valorized as protein isolates, animal feed, and waste-derived ingredients with high nutritional value. Further, drying and coating technologies are enhancing the shelf life and stability of chayote-based products, further encouraging wider use as a sustainable and functional food ingredient in the food industry.

### 7.1. Fermented and Functional Products

Fermentation appears to enhance the nutritional and functional value of chayote by increasing its bioactive potential. During large-scale natural fermentation used for pickled chayote production, *Lactobacillus alimentarius* was identified as the predominant bacterial species, whereas *Kazachstania humilis* and *Pichia membranifaciens* were the main yeast species. Microbial metabolic network analysis showed that both bacterial and fungal communities work together to shape physicochemical properties and generate characteristic flavor compounds [20]. Through combined genomic and metabolomic analysis, *L. plantarum* was identified as the major source of metabolic genes and volatile metabolites, including aldehydes, esters, and alcohols, mainly through phenylalanine metabolism, the Ehrlich pathway, and fatty-acid biosynthesis pathways [19]. In another study, LAB fermentation of chayote leaf-pineapple smoothies increased total phenolic recovery by 65.96% during simulated intestinal digestion, while dialysis-based bioavailability reached 53.58% [101]. Additionally, a folate-producing probiotic strain, *Lactiplantibacillus plantarum* MGKMVIT11, isolated from fermented chayote peel, produced 364 µg/mL folate under optimized conditions and demonstrated antioxidant, anti-inflammatory, and anticancer activity against HCT-116 cells [102].

### 7.2. Pectin Extraction and Functional Properties

Chayote pectin has emerged as a high-value functional ingredient. UAE-optimized extraction yielded 6.19% pectin with a low degree of esterification, high molar mass (2.47 × 10^6^ g/mol), non-Newtonian shear-thinning behavior, and ABTS antioxidant capacity exceeding that of commercial apple pectin [15]. Structural analysis by FTIR, methylation analysis, and NMR characterized two cell-wall polysaccharide fractions: CP-1 (galactan, 41.1 kDa) and CP-2 (anionic heteropolysaccharide, 15.6 kDa) with a complex branched backbone incorporating galacturonic acid, galactose, arabinose, rhamnose, glucose, glucuronic acid, mannose, and xylose [45,64]. The gut-regulatory activity of chayote pectin—via PI3K/AKT/FoxO1 and GPR43/AMPK/FoxO1 axes—positions it as a dietary fiber with prebiotic and antidiabetic functionality beyond rheological applications [10].

### 7.3. Starch-Based Films and Biodegradable Packaging

Chayote tuber starch (CTS) has gained attention as a promising raw material for biodegradable food packaging applications. Composite CTS films incorporating cinnamon essential oil (CEO) Pickering emulsion stabilized with zein-pectin complexes (ZPCO) showed greater elongation at break, improved antimicrobial activity against foodborne pathogens, enhanced antioxidant potential, and lower water vapor permeability at 2% ZPCO. These films also enabled slow and sustained release of CEO and noticeably prolonged the shelf life of ground beef without showing toxic effects at the tested concentrations [16]. Similarly, the addition of cellulose nanoparticles to CTS/potato starch blends strengthened the films by increasing tensile strength, elastic modulus, melting temperature, and enthalpy, while also lowering solubility [17]. Reactive extrusion (REX)-based cationization of chayote root starch using 3% glycidyltrimethylammonium chloride (GTAC) successfully modified its physicochemical properties, while offering a faster, more energy-efficient, and environmentally favorable alternative to conventional cationization [103]. In addition, biopolymer films prepared from immature chayote peel and plasticized with glycerol, sorbitol, or ethylene glycol exhibited a glass transition temperature of 63.29 °C and a melting temperature of 154.26 °C, indicating thermal stability appropriate for potential food packaging use [55]. Luna-Niño et al. [104] developed biodegradable chayotextle starch films supplemented with rosemary or cinnamon essential oils, which exhibited antimicrobial and antifungal activity and showed water-activity-dependent mechanical properties. When applied as edible coatings to bread, these films delayed mold growth during 15 days of storage while maintaining acceptable sensory characteristics, demonstrating their potential to extend bread shelf life.

### 7.4. Protein Isolates and Value-Added Ingredients

Chayote seeds are a relatively underexplored nutraceutical source. Protein yield recovery was enhanced to 88.0 ± 3.2% using a 20 kHz ultrasound probe, and a protein content of 8.2 ± 0.9% dry weight was obtained in a chayote seed protein isolate (CSPI). The essential amino acid content of the resulting CSPI had a desirable score of 315.63 mg/g protein; its protein digestibility was 80.3 ± 4.5%, while the α-amylase inhibitory activity was 74% at 100 µg/mL [19]. CSPI-UAE-20 kHz also had the highest antioxidant capacity, with a TPC of 7.22 mg GAE/g DW, suggesting that seed-derived proteins function as both functional food components and bioactive agents. Chayote shoots and leaves, characterized as waste fractions, contain high concentrations of myricetin and catechin, with the highest antioxidant activity of all plant parts, and elevated minerals (Ca, K, Mg, P, Fe, Zn), presenting circular economy opportunities for by-product valorization [32]. Chayote meal (fruit and leaves combined at 4:1) safely replaced standard pig grower ration at levels of up to 40% in growing indigenous Zovawk pigs, with no significant adverse effects on average daily gain (ADG approximately 0.18–0.24 kg), feed conversion ratio, or nutrient digestibility of dry matter, crude protein, or crude fiber, demonstrating its potential as an alternative animal feed ingredient [105]. Purees from discarded chayote retail waste showed high nutritional value (phenolics, fiber) and were microbiologically safe in 90% of cases, suggesting their viability for new product development [106].

### 7.5. Drying, Coating, and Shelf-Life Technologies

**Figure 2:**
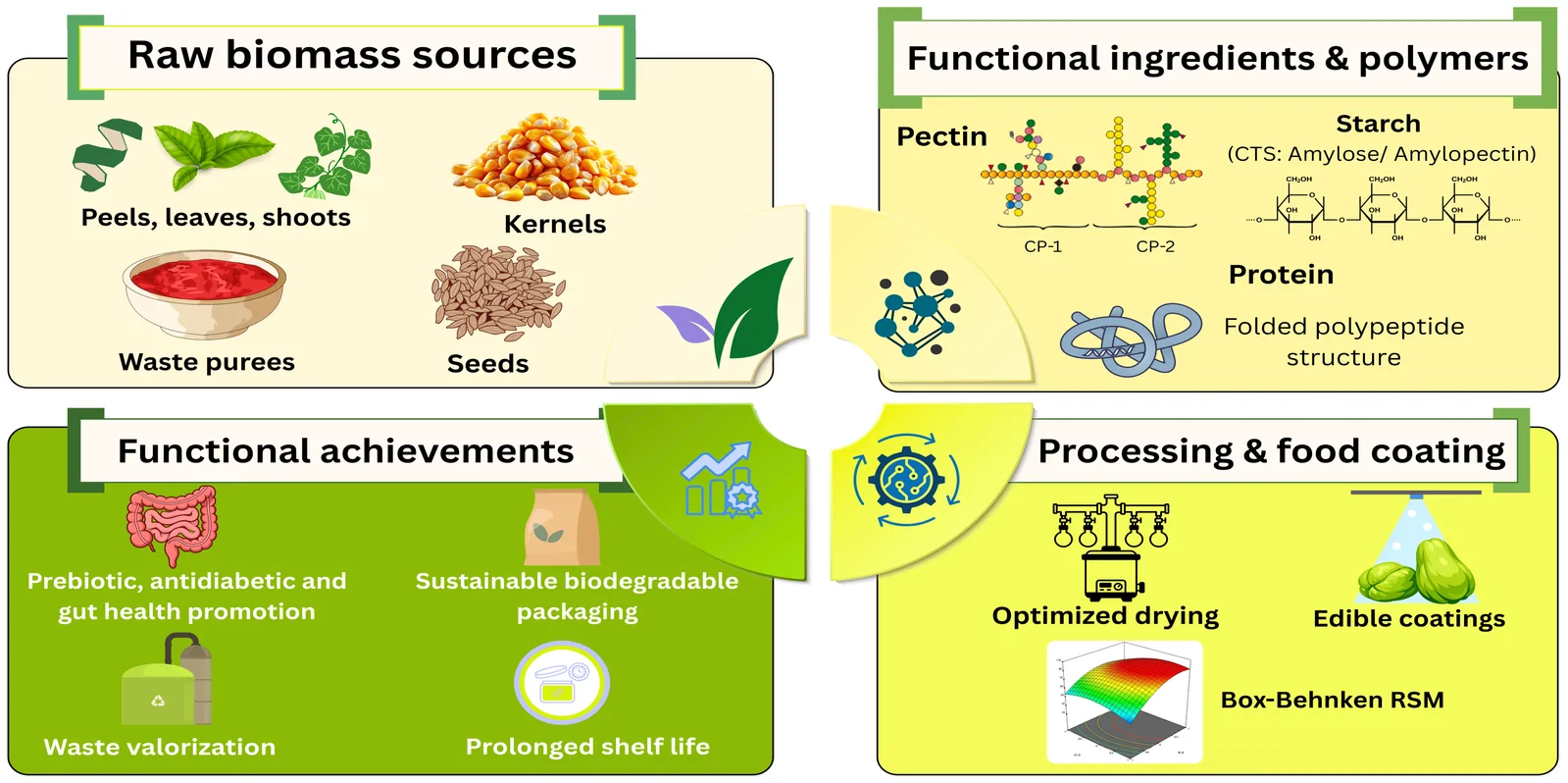
Food-industry applications of *S. edule*, converting biomass by-products into functional food ingredients. Raw biomass sources (peels, leaves, shoots, kernels, waste purees, and seeds) are processed into functional polymers, including pectin (fractions CP-1 and CP-2), starch (composed of amylose/amylopectin) and protein, which are further engineered through optimized drying and edible coating formulations using Box–Behnken response surface methodology (RSM) [8,13,14,15,43,68,103,104,105,106].

Composite edible coatings (pectin, soy protein isolate, xanthan gum) optimized by Box–Behnken RSM improved the moisture content, water activity, and rehydration properties of convective-dried chayote slices, with the parabolic model providing the best fit for thin-layer drying kinetics (R^2^ 0.996–0.999) [107]. Sorption isotherms exhibited Type IV behavior best described by the BET model, providing critical data for optimizing storage stability. These findings support the industrial-scale application of coated dried chayote slices as stable, nutritious convenience products. The applications of *S. edule* are shown in Figure 2.

## 8. Safety and Toxicity

The edible fruit of chayote is traditionally eaten in several parts of the world, fresh, cooked, or processed [1,9]. However, this does not apply to extracts, purified phytochemicals, non-food plant parts, or products taken at pharmacologic dosages. Toxicological evidence for *S. edule* is limited compared with its biological and therapeutic activities.

The most detailed acute toxicity study was conducted with the methanolic extract of fruits of *S. edule* var. *nigrum spinosum*. No mortality was recorded in CD-1 mice given doses ranging from 8 to 5000 mg/kg body weight, and no significant changes in blood chemistry or peripheral blood cell counts were found at the highest dose tested, although an increase in the mitotic index of bone marrow cells was observed. The extract also showed significantly higher cytotoxic activity against P388 leukemia cells than against normal mononuclear bone marrow cells, indicating some biological selectivity under the experimental conditions described above [44]. These results show that the methanolic extract of the fruits is not acutely toxic but do not represent a long-term or repeated-exposure safety assessment because of the short duration of the observations and the fact that the extract was prepared experimentally.

Preliminary genotoxicity investigations were also conducted. After two weeks of fresh chayote juice consumption, the filtered and unfiltered fresh chayote juices were assessed in mice by a peripheral-blood micronucleus assay. Juice from both preparations was not genotoxic under the tested conditions, and both juices were found to be antigenotoxic against benzo[a]pyrene-induced DNA damage [83]. The results provide further confidence in the tested fresh juice preparations but are not necessarily applicable to other extracts from *S. edule* or its purified constituents. Specifically, genotoxicity testing according to international guidelines remains lacking for concentrated extracts, different plant parts, extended exposure times, and extracts enriched in specific secondary metabolites.

Additionally, human studies provide indirect information on tolerability. The powdered form of dried fruit of *S. edule* var. *nigrum spinosum* has been used in older adults with MetS or T2DM for several weeks to 6 months, with doses of 500 mg three times a day (1.5 g/day) [8,13,76,82]. The studies focused mainly on metabolic, inflammatory, antioxidant, telomere-related, Nrf2-mediated, and sirtuin-related outcomes and not safety as a primary endpoint. No apparent toxicity signal is evident from the published interventions, although the published trials are generally small, short-term, and were not primarily designed to assess adverse events, organ toxicity, dose–response relationships, or long-term clinical safety.

The absence of toxicity reports in these studies should not be taken as definitive evidence of long-term therapeutic safety. Concentrated preparations are especially important from a safety standpoint because chayote contains secondary metabolites with pharmacological activity, such as cucurbitacins. Pharmacokinetic studies in mice showed systemic exposure to several polyphenols and cucurbitacins after oral administration of a methanolic extract of the H387 07 *Sechium* hybrid at doses of 8, 125, and 250 mg/kg [41]. Cucurbitacin B showed a peak concentration (Cmax) of 37.56 µg/mL at 1 h after its administration at a dose of 125 mg/kg, indicating rapid absorption, and other compounds such as cucurbitacin IIA, apigenin, and phloretin were also found in biological samples [41]. These observations reflect systemic bioavailability but not necessarily safety. Accumulation, metabolism, or tissue-specific exposure may occur with repeated administration and should be determined by repeated-dose pharmacokinetic studies, especially for concentrated preparations containing cucurbitacin.

Environmental and agricultural conditions are also crucial for food safety. Chayote was found to be one of the relatively low heavy metal-accumulating vegetables in farmland near a phosphorus chemical plant, and the exposure scenario used in this study showed that consuming chayote does not pose a carcinogenic or non-carcinogenic risk compared with the other vegetables studied [108]. These results are encouraging but apply only to the soil environment and irrigation system investigated and do not rule out contamination risk in other soil, irrigation, or industrial conditions. In addition, chayote was added to the list of fruit and vegetable matrices for which a GC-MS method was validated for dithiocarbamate fungicide residues, with overall recoveries of 75–104% [109]. In the above-mentioned study, however, the removal of mancozeb residues from vegetables during cooking was not specifically measured in chayote, so conclusions about the effects of cooking on reducing residues in chayote are limited.

The microbiological risks from the manufacturing environment should also be considered. In agricultural lands where large amounts of poultry litter are applied as fertilizer, antimicrobial-resistant bacteria such as clinically relevant *Enterobacteriaceae* and various antimicrobial-resistance genes were found in poultry litter, cultivated soil, the rhizosphere of *S. edule*, irrigation water, and sediments [110]. Notably, in that investigation, edible chayote fruits were not directly sampled, and the study illustrates an environmental pathway that can spread antimicrobial resistance within chayote production systems without directly contaminating the edible product. The results also highlight the need for adequate manure management, water quality in irrigation systems, hygiene in agricultural production, and monitoring of antimicrobial resistance in production systems.

Another risk area that has not been well studied is potential herb–drug interactions. Chayote was listed among the herbs commonly used by Latino/Hispanic adults with T2DM to manage their diabetes [111]. The same study found that 77% of those taking herbal remedies were also taking prescribed medicines at the time, and 77% of these individuals did not mention herbal remedies to their healthcare providers; some said they changed or even discontinued prescribed diabetes medicines to use the herbal medicines [111]. These percentages apply to all users of herbal remedies, not chayote users specifically, but they indicate the need to study potential pharmacodynamic and pharmacokinetic interactions with antidiabetic, antihypertensive, anticoagulant, and other frequently prescribed medications.

Available evidence indicates a favorable safety profile for traditionally consumed chayote fruit and the specific preparations studied so far, although evidence remains insufficient to confirm the safety of long-term use of concentrated chayote products (*S. edule*). There are several significant gaps in the research, such as repeated-dose and subchronic toxicity studies (standardized), reproductive and developmental toxicity, more extensive genotoxicity testing across different plant parts and extraction methods, repeated-dose and long-term pharmacokinetic studies, systematic monitoring of adverse events in larger RCTs, and direct assessment of herb–drug interactions. Particular attention should also be given to purified or cucurbitacin-enriched fractions and other highly concentrated formulations, for which safety cannot be inferred from traditional dietary consumption of the fruit. Consequently, safety and therapeutic conclusions should remain specific to the plant material, extraction method, dose, route of administration, and duration of exposure actually evaluated in each study.

## 9. Integrated Mechanistic Insights

The diverse biological activities of *S. edule* converge on several interconnected molecular mechanisms. Phenolic compounds and flavonoids reduce oxidative stress by scavenging reactive oxygen species (ROS) and reactive nitrogen species (RNS) and by activating Nrf2-mediated expression of antioxidant genes (SOD, CAT, GPx, and HO-1) [83]. Chayote water-soluble polysaccharides have been shown to inhibit inflammatory responses in THP-1 macrophage-like cells pretreated with cholesterol crystals by downregulating the release of IL-1β and chemokines, caspase-1 activation, and NLRP3 and IL-1β gene expression, suggesting that they inhibit the priming of the NLRP3 inflammasome [46]. Oxidative stress can also contribute to NF-κB-mediated activation of the NLRP3 inflammasome pathway: ROS scavenging has been shown to inhibit NF-κB and NLRP3 activation and the cytokine release associated with inflammasome activation [112]. In metabolic disorders, pectin-derived short-chain fatty acids (SCFAs), generated through gut microbial fermentation, activate G-protein-coupled receptor 43 (GPR43) and AMP-activated protein kinase (AMPK). AMPK subsequently phosphorylates and inactivates forkhead box protein O1 (FoxO1), thereby suppressing the expression of key gluconeogenic enzymes, including glucose-6-phosphatase (G6Pase) and phosphoenolpyruvate carboxykinase (PEPCK), resulting in improved glucose homeostasis. In parallel, alterations in gut microbiota composition enhance glucagon-like peptide-1 (GLP-1) secretion, which activates the phosphatidylinositol 3-kinase/protein kinase B (PI3K/Akt) signaling pathway and further improves glucose metabolism [10]. Upregulation of sirtuins (SIRT1, SIRT3, SIRT5, and SIRT6) by chayote bioactives integrates antioxidant, metabolic, and aging-related pathways [84]. Cucurbitacins (primarily cucurbitacins B, D, E, and I) and flavonoids, including quercetin, myricetin, and naringenin, induce apoptosis in tumor cells through the intrinsic mitochondrial pathway by promoting cytochrome c release and caspase-3/9 activation, with lower toxicity toward normal cells reported in some experimental models [43,92,94]. Geroprotective effects, including telomere maintenance and sirtuin activation, likely emerge from the combined antioxidant, anti-inflammatory, and metabolic regulatory activities of chayote polyphenols and dietary fiber [84,100].

## 10. Cultivation Constraints, Pathogens, and Pest Management

Chayote production is affected by various biotic stressors. Fungal and oomycete diseases include anthracnose caused by several species of *Colletotrichum*, such as *C. chrysophilum*, *C. menezesiae*, *C. plurivorum*, *C. karsti*, the recently described *C. cucurbitacearum* and *C. sicyi* [113]. In cross-pathogenicity tests, onion leaves were susceptible to a *C. gloeosporioides* isolate, and isolates from chayote and other hosts were pathogenic to onion leaves [114]. Other reported diseases include powdery mildew, associated with *Podosphaera xanthii* [115] and *Neoerysiphe sechii* [84], whereas *Didymella bryoniae* [116] is the cause of gummy stem blight, forming the new family Pleosporales, genus *Leandriaceae*, *Leandria momordicae*, the cause of net spot [117], and *Phytophthora capsici*, the cause of plant wilt and fruit rot.

Viral pathogens such as ToLCNDV have been reported in southern India [118], whereas a different strain of ToLCNDV reported from Tamil Nadu resulted in 50.3–100% seed transmission and disease incidence [119]. Squash leaf curl Philippines virus has been reported infecting chayote in Taiwan [120], and Pepper golden mosaic virus was found in chayote in Costa Rica [121]. ToLCNDV strains from India (chayote) and Spain (Mediterranean) differed in their vector competence, with efficient transmission by *T. vaporariorum* of the chayote strain of ToLCNDV, while this whitefly species was not able to transmit the strain associated with Spain (Mediterranean) [118,122]. Insect pests and associated herbivores are the melon fly *Bactrocera cucurbitae*, managed using bait stations adapted to chayote trellises [123], the cucurbit fruit fly *Anastrepha grandis* [124], the treehopper *Campylocentrus nigris* which is associated with chayote and parasitized by the braconid *Nealiolus chayohtli* [125], and the stem-boring weevil *Phymatophosus squameus*. Visible/near-infrared (Vis/NIR) spectroscopy has improved nondestructive quality assessment of chayote. A multi-product calibration model for six Cucurbitaceae commodities, including chayote, was developed with prediction coefficients (R^2^p) of 0.96 for soluble solids content and 0.92 for water content [126], and an enhanced Vis/NIR spectral dataset was later released to promote additional model development [127]. The GARP-ARR-B transcription factor SeAPRR2 has been identified as a promising molecular target for enhancing drought tolerance in chayote germplasm through genetic improvement [128].

## 11. Cultural Significance, Food Security, and Societal Dimensions

Chayote holds an important place in the food cultures of Latin America, Southeast Asia, and Caribbean communities. In addition, chayote is a culturally specific vegetable in Latino food environments in the U.S. A survey of retail food stores in majority-Latino communities and majority-African American communities in southwest Chicago found that grocery stores in Latino communities were significantly more likely to carry chayote than grocery stores in African American communities (82.0% vs. 17.6%, *p* < 0.05) [129].

Culturally significant vegetables purchased through fruit and vegetable vouchers by rural Mexican-heritage households participating in the WIC program often included chayote [130]. Sixty-nine percent of Latino/Hispanic adults with T2DM in North Carolina said they use herbal remedies as part of their diabetes care, with chayote being one of the four most frequently mentioned remedies along with prickly pear cactus, aloe vera, and celery. Of those who used herbal remedies, 77% said they used them alongside prescribed medication, and 77% did not tell their healthcare providers about this [111]. In Jamaica, mothers recognized chayote (“chocho”) as a culturally appropriate complementary food for babies [131]. Among indigenous vegetables, those with higher nutrient density were consumed by school-aged adolescents in the Philippines as part of overall vegetable intake; dietary fiber and various minerals were generally linked to the normal BMI category [132]. Chayote was included among foods consumed nonhabitually (less than four times per week), and the diet of the population analyzed was found to have gaps that may increase the risk of diet-related chronic diseases in rural Brazil [133]. These diverse contexts underscore chayote’s potential as a food security crop that can contribute to nutritional diversity across different demographic settings.

## 12. Limitations and Future Perspectives

Several overarching limitations constrain the translation of existing evidence into clinical and industrial applications. First, most bioactivity studies rely on in vitro assays and animal models using standardized extracts that may not reflect dietary consumption. Only a limited number of clinical trials have been conducted, primarily in Mexican cohorts of older adults with MetS or T2DM, limiting generalizability [12,13,82,84]. Second, phytochemical composition varies widely across cultivars, plant parts, maturity stages, geographical origins, and extraction solvents, making cross-study comparisons difficult [1,18]. Third, pharmacokinetic and bioavailability data remain sparse: while rapid absorption of cucurbitacin B was documented [41], systematic absorption, distribution, metabolism, and excretion (ADME) profiles for key polyphenols and fiber fractions are lacking.

Future research priorities include: (1) randomized controlled trials in diverse populations with standardized extract preparation and dose–response characterization, (2) bioavailability and pharmacokinetics of chayote polyphenols and pectin in healthy volunteers, (3) chronic safety assessment (OECD 28-day and 90-day repeated-dose toxicity; genotoxicity; reproductive toxicity) of concentrated extracts, (4) mechanistic studies on telomere maintenance and sirtuin activation in aging populations, (5) molecular breeding programs leveraging the sequenced genome and SSR markers to develop cultivars enriched in specific bioactives, (6) scale-up validation of UAE-extracted pectin, seed protein isolates, and starch-based biodegradable films for commercial food and packaging applications, (7) microbiome–bioactivity relationship studies exploring how chayote fiber and polyphenols modulate gut microbiota composition and metabolic function in human subjects, and (8) integrated food system analyses evaluating postharvest management, cold chain requirements, and sustainable packaging solutions to reduce the approximately 15–30% postharvest losses affecting chayote supply chains [26,107]. Beyond bioactivity validation, translating chayote-derived phytochemicals into marketable therapeutics will also require navigating patent linkage and regulatory-exclusivity frameworks, as such systems can significantly influence the pace and cost of natural-product drug development [134].

## 13. Conclusions

*S. edule* represents a scientifically compelling, culturally significant, and commercially underexplored species with multifaceted potential for food, nutraceutical, and pharmaceutical development. Its diverse phytochemical portfolio, spanning flavonoids, phenolic acids, cucurbitacins, pectins, polysaccharides, carotenoids, and seed proteins, underpins a broad range of validated biological activities, with emerging clinical evidence supporting antioxidant, hypoglycemic, and anti-inflammatory effects in human subjects. The mechanistic integration of Nrf2 activation, sirtuin upregulation, PI3K/AKT/FoxO1 and GPR43/AMPK/FoxO1 signaling, NLRP3 inflammasome suppression, and intrinsic apoptotic pathway activation in tumor cells positions chayote as a functional food with multitarget health-promoting potential. From an industrial perspective, chayote’s tuber starch, peel, seed, pectin, and fermented fractions offer concrete opportunities for biodegradable packaging, functional ingredients, bioactive films, and probiotic food development, supported by published process optimization data. Advances in genomics (chromosome-level assembly, domestication history, SSR markers, and transcription factor characterization) are laying the groundwork for precision breeding of bioactive-enriched cultivars. Strategic investment in standardized clinical research, bioavailability characterization, safety documentation, and circular processing of underutilized plant fractions will be essential to fully realize the potential of this neglected crop in global food and health systems. Despite these promising findings, important limitations remain, including the lack of large multicenter randomized controlled trials, variability in phytochemical composition among chayote genotypes and extracts, limited data on absorption, distribution, metabolism, and excretion (ADME), and insufficient long-term safety evaluation of concentrated chayote extracts.

## Figures and Tables

**Table 1:** Nutritional composition, antioxidant activity, and major bioactive compounds identified in different parts of *S. edule*.

Plant Parts	Proximate Composition (%)	Vitamins (mg/100 g)	Minerals (mg/100 g)	Amino Acids (mg/g Protein)	Antioxidant Activity	Bioactive Compounds	References
Seeds	Moisture: 77.56; crude protein: 5.5; crude fat: 0.4; carbohydrates: 60; fiber: 2.41; ash content:3.58; starch: 1.9; total sugars: 4.2	NR	Calcium: 68.0; phosphorus: 312.0; iron: 4.2	Arginine: 135.40; glutamic acid: 65.70; lysine: 46.80; valine: 46.80; aspartic acid: 34.70; serine: 31.10; leucine: 2.69; phenylalanine: 1.81; isoleucine:1.30; threonine: 0.88; histidine: 0.67	ABTS: 28%; antioxidant activity (tincture extract): 76%	TPC: 2.1 mg GAE/g (dw); trypsin inhibitors; DPPH assay (IC_50_ ≈ 2 μg/mL)	[18,48,49,50]
Leaves	Crude protein: 2.69; crude fat: 2.32	Vit C: 4.6	NR	Glutamic acid: 12.15; alanine: 11.36; glycine: 11.01; leucine: 9.89; valine: 8.34;	DPPH IC_50_: 0.5 mg/mL; ABTS IC_50_: 0.7 mg/mL; FRAP: 22.3 μM Fe(II)/g; antioxidant activity (fluid extract): 91%	TPC: 89.3 mg CE/g; TFC: 67.2 mg QE/g; total carotenoids: 4.5 mg/100 g; anthraquinones; phenolic acids; coumarins, anthocyanins; apigenin 6-C-β-D-glucopyranosyl-8-C-β-D-apiofuranoside; diosmetin 7-O-rutinoside; luteolin 7-O-rutinoside (0.141 g/100 g, DW); trans-cinnamic acid, phenylacetic acid, 3-octadecenoic acid, trilinolenin, α-linolenic acid	[47,48,49,50,51]
Tuberous roots	Moisture: 82.3; crude protein: 2.0; crude fat: 0.2; carbohydrates: 17.8; fiber: 0.4; ash content: 1.0; starch: 66.9; total sugars: 9.0	Vit C: 19; thiamine: 0.05; riboflavin: 0.03; niacin: 0.90	Phosphorus: 34; calcium: 7.0; iron: 0.8	NR	NR	Cinnamic acid methyl ester; anthraquinones; phenolic acids; coumarins, anthocyanins; apigenin 6-C-β-D-glucopyranosyl-8-C-β-D-apiofuranoside; diosmetin 7-O-rutinoside; vitexin	[52,53]
Stems	Moisture: 90.29; crude protein: 3.15; crude fat: 0.19; carbohydrates: 3.20; fiber: 1.87; ash content: 1.17; starch: 0.70; total sugars: 0.30	Vit E: 90; vit C: 16; thiamin: 0.08; riboflavin: 0.18; niacin: 1.10; vit A: 615 IU/100 g	Phosphorus: 20.6; calcium: 13.1; magnesium: 6.6; sodium: 0.4; copper: 0.03; iron: 0.36	NR	DPPH: 255.92 μg/mL; antioxidant activity (tincture extract): 65%	TPC: 350.38 mg/g GAE; anthraquinones; phenolic acids; coumarins, anthocyanins	[48,54]
Fruit peel	Moisture: 35; crude protein: 15.5; crude fat: 0.064; fiber: 45.2; ash content: 7.59; starch: 0.368;	Vit C: 51.6	Calcium: 307; iron: 6.76;	NR	DPPH IC_50_: 0.4 mg/mL; ABTS IC_50_: 0.7 mg/mL; FRAP: 17.4 μM Fe(II)/g	TPC: 66.0 mg GAE/g; TFC: 48.6 mg QE/g; total carotenoids: 9.7 mg/100 g; β-carotene: 0.36 mg/100 g; cinnamic acid methyl ester; coumaric acid; vitexin	[50,55,56,57,58]
Fruit pulp	Moisture: 93.2; crude protein: 0.84; crude fat: 0.15; carbohydrates: 4.42; crude fiber: 0.27; ash content: 0.3; starch: 10.9; total sugars: 22.1	Vit C: 4.95–7.82; thiamin: 0.03; riboflavin: 0.03; niacin: 0.47; pantothenic acid: 0.25; pyridoxine: 0.08; folic acid: 0.093; vit E: 0.12; vit K: 0.41; vit A: 56 IU/100 g	Phosphorus: 100; potassium: 300; calcium: 40; magnesium: 60; zinc: 55; iron: 3.1; copper: 0.4; nitrogen: 0.5; manganese: 0.5	Glutamic acid: 1.97; aspartic acid: 1.45; leucine: 1.21; valine: 0.99; alanine: 0.80	DPPH IC_50_: 0.7 mg/mL; ABTS IC_50_: 0.1 mg/mL; FRAP: 8.3 μM Fe(II)/g	Cucurbitacins: 2.7–66 mg/100 g; TPC: 21.27 mg GAE/100 g; TFC: 4.64 mg CE/100 g; total carotenoids: 8.3 mg/100 g; cinnamic acid methyl ester; coumaric acid; vitexin	[40,44,59,60,61]

Abbreviations: ABTS, 2,2′-azino-bis(3-ethylbenzothiazoline-6-sulfonic acid); CE, catechin equivalents; DPPH, 2,2-diphenyl-1-picrylhydrazyl; DW, dry weight basis; FRAP, ferric reducing antioxidant power; GAE, gallic acid equivalents; IC_50_, half-maximal inhibitory concentration; IU, international unit; NR, not reported; TFC, total flavonoid content; TPC, total phenolic content; Vit, vitamin.

**Table 2:** Green extraction methods versus traditional methods, phytochemical profiles, and bioactive potential of *S. edule* extracts.

Plant Part	Extraction Method	Solvent Used	Optimized Conditions	Extract Yield (%)	Major Phytochemicals/Extract Properties	References
Fruit peel	UAE, microwave-assisted extraction (MAE), and maceration comparison	Hydroethanolic solvent system	Chayote peels were extracted using UAE, MAE, and conventional maceration. Extracts were evaluated for phenolics, carotenoids, antioxidant capacity, and cosmetic potential.	UAE: 9.34%; MAE: 6.56%; ME: 6.88%	Total phenolic compounds, carotenoids and other antioxidant phytochemicals. UAE provided the highest recovery of bioactive compounds; UAE peel extract showed superior antioxidant performance compared with MAE and maceration. The extract showed low cytotoxicity in HaCaT keratinocyte cells and was incorporated into a stable gel formulation for cosmeceutical application.	[63]
Fruit pulp	Conventional hot-water extraction after enzymatic starch removal	Acidified distilled water	Chayote powder was enzymatically treated with α-amylase (4000 U/g, pH 6.0, 60 °C, 30 min) to remove starch. Residue was extracted with water (1:50 g/mL, pH 2) at 70 °C for 100 min. Polysaccharides were precipitated with ethanol and purified.	NR	Non-starch polysaccharides are composed mainly of galactan, arabinogalactan, homogalacturonan and rhamnogalacturonan-type structures; the extract exhibited desirable physicochemical properties, including water-holding ability and potential application as a functional food polysaccharide.	[64]
Fruit, leaves, and pedicel	Reflux solvent extraction (polarity-based fractionation)	Different polarity solvents: hexane, ethyl acetate, ethanol, and water	Plant organs were extracted using a reflux apparatus with solvents of different polarity. Extracts were concentrated using rotary evaporation and analyzed for phytochemicals and antioxidant capacity.	NR	Ethyl acetate fruit extract showed the highest phenolic content (3.21 g GAE/100 g extract). Ethyl acetate leaf extract showed the highest flavonoid content (11.64 g QE/100 g extract) and carotenoid content (12.73 g β-carotene equivalents/100 g extract). Ethyl acetate pedicel extract showed the strongest DPPH activity (IC_50_ = 1.3 µg/mL), while ethyl acetate fruit extract exhibited the strongest CUPRAC activity (EC_50_ = 147 µg/mL).	[65]
Fruit pulp and leaves	Sequential solvent extraction (fractionation approach)	Methanol and ethyl acetate fractions after sequential extraction	The dried sample was extracted sequentially to obtain phenolic-rich fractions. The methanolic extract was further partitioned with ethyl acetate to enrich phenolic compounds.	NR	The ethyl acetate fraction showed the highest antioxidant activity, with a DPPH radical scavenging capacity of 951.73 ± 29.14 mM Trolox equivalents/g extract. Strong correlation (r = 0.99) was observed between TPC and antioxidant activity. Extracts also showed α-glucosidase inhibitory potential.	[66]
Tuberous roots	Hydroalcoholic maceration	Ethanol: water (60:40, *v*/*v*)	Roots were dried, milled, and extracted with hydroalcoholic solvent. The extract was concentrated and lyophilized.	~8.9% dry extract recovery	Phenolic acids, flavonoids, and cinnamic acid derivatives; the extract exhibited antioxidant activity and vascular protective effects associated with antihypertensive activity.	[53]
Tuberous roots	Conventional hydroalcoholic maceration	Ethanol: water (3:2, *v*/*v*)	Fresh roots dried at 50 °C for 72 h, ground (3–5 mm), macerated for 24 h, filtered, vacuum concentrated, and lyophilized	712 g dry extract from 8 kg dried roots (≈8.9% yield)	Hydroalcoholic extract exhibited potent antihypertensive and vasorelaxant activities. The active fraction was rich in cinnamic acid derivatives (e.g., cinnamic acid methyl ester), phenolic compounds, and coumaric acid derivatives.	[67]
Tuberous root	Hydroalcoholic extraction	Ethanol: water (70:30, *v*/*v*)	Dried roots were powdered and extracted by hydroalcoholic maceration. The extract was filtered, concentrated, and evaluated in a hepatocyte steatosis model.	8.90% (calculated from 712 g dry extract obtained from 8 kg dried plant material)	Phenolic acids, especially cinnamic acid and coumaric acid derivatives; the extract reduced oxidative stress markers, decreased triglyceride accumulation, improved AMPK activation, and reduced inflammatory responses in an oleic acid-induced steatosis model.	[68]
Leaves	Conventional maceration (ME), UAE, and microwave-assisted extraction (MAE)	50% ethanol–water	Solid-to-solvent ratio 1:30 (g/mL); 55 °C for 30 min; UAE: 170 W; MAE: 300 W	ME: 7.18 ± 1.02%; UAE: 11.80 ± 1.32%; MAE: 9.01 ± 1.70%	UAE produced the highest extraction yield and was most effective for recovering bioactive compounds, including total phenolics (5.38 ± 0.28 mg GAE/g DW), carotenoids (0.85 ± 0.02 mg/g DW), flavonoids (myricetin, rutin, quercetin), phenolic acids (chlorogenic and ferulic acids), β-carotene, xanthophylls, α-tocopherol, and exhibited superior ABTS and FRAP antioxidant activities.	[18]
Fruit peel, leaves, and pulp	Conventional solvent extraction	Methanol extraction followed by evaluation of fresh and cooked extracts	Plant tissues were extracted with methanol to recover phenolic compounds. Fresh samples and processed samples (roasted and steamed) were compared for changes in bioactive compounds.	Peel = 2.9%; Leaves = 2.5%; Pulp = 1.7%	Fresh pulp showed the highest phenolic content (58.5 mg/g extract). Peel showed the strongest DPPH radical scavenging activity (IC_50_ = 0.4 mg/mL), while pulp exhibited the highest ABTS activity (IC_50_ = 0.1 mg/mL). Peel extract showed strong α-amylase inhibitory activity (IC_50_ = 0.2 mg/mL). Processing reduced phenolic content and bioactivity in most cases.	[50]
Fruit	Soxhlet extraction and maceration	Petroleum ether defatting followed by ethanol extraction	Soxhlet extraction with petroleum ether for 6 h; residue extracted by ethanol maceration for 24 h. TLC used for compound identification.	NR	Alkaloids, saponins, cardenolides/bufadienolides, flavonoids; Demonstrated presence of secondary metabolites in ethanol fruit extract.	[69]
Whole fruit (cv. Madre negra)	Sequential solvent extraction and chromatographic fingerprinting	Hexane→dichloromethane→methanol	Dried whole fruits (exocarp + mesocarp + seed) were powdered. Sequential extraction was performed with each solvent for 48 h at room temperature. Extracts were concentrated and analyzed by TLC and chromatographic separation for cucurbitacins.	NR	Cucurbitacins and other non-polar to medium-polar secondary metabolites; fractions were characterized by chromatographic fingerprinting; Extract fractions demonstrated antiproliferative activity against breast cancer cell models, with activity associated with specialized metabolites rather than only phenolic compounds.	[70]
Fruit	Methanolic extraction followed by TLC, column chromatography and HPLC fractionation	Methanol	Fruit material was extracted with methanol; crude extract was fractionated into terpene and flavonoid fractions.	7.48% methanolic extract yield (78.302 g from 1.13 kg sample)	Terpenes: cucurbitacins B, D, E and I; flavonoids: rutin, myricetin, quercetin, naringenin, phloretin, apigenin, galangin; Methanolic extract and fractions showed antiproliferative activity against HeLa cells.	[43]
Seed	Ultrasound-assisted alkaline extraction (probe sonication, UAE-20 kHz)	Distilled water (alkaline medium; pH adjusted to 11 with 2 M NaOH, protein precipitated at pH 4.0 with 1 M HCl)	10% (*w*/*v*) seed suspension; 20 kHz, 500 W, 25% amplitude; 20 min; temperature maintained below 10 °C using an ice bath	Protein Recovery Alkaline extraction: 58%; Ultrasound probe: 88%; Ultrasound bath: 72.4%	Highest protein purity (66.7%), balanced amino acid profile, essential amino acids (315.63 mg/g protein), protein digestibility (80.3%), highest total phenolics (7.22 mg GAE/g DW), strong antioxidant activity and 74% α-amylase inhibition	[19]
Fruit (cell-wall fraction)	Ultrasound-assisted acidic aqueous extraction followed by ethanol precipitation and chromatographic purification	Acidified water (pH 2)	Chayote powder was first treated with α-amylase to remove starch. The residue was extracted with water (solid: liquid ratio 1:50 g/mL) under ultrasound treatment (180 W, 70 °C, 40 min). Extracted polysaccharides were precipitated using two volumes of ethanol and purified by DEAE-52 cellulose and Sephadex-200 chromatography.	Fraction yields after purification: CP-1: 9.2%, CP-2: 12.6% (from crude fractions)	Two purified polysaccharides: CP-1 (41.1 kDa galactan) and CP-2 (15.6 kDa acidic heteropolysaccharide) containing galacturonic acid, galactose, arabinose, rhamnose, glucose, glucuronic acid, mannose and xylose; Demonstrated physicochemical functionality suitable for food applications. The polysaccharides showed excellent structural stability, water interaction properties, and potential as functional food polysaccharide ingredients.	[45]
Fruit (hybrid and segregant lines)	Methanolic extraction followed by chromatographic phytochemical characterization	Methanol	Fruit tissues from different *S. edule* genetic materials were extracted with methanol and analyzed for phytochemical composition and pharmacological potential.	NR	Total cucurbitacins = 8.25 mg/g extract; Flavonoids, phenolic compounds, and secondary metabolites; detailed phytochemical profiling was performed for H387-07 and H387-M16 extracts; Extracts demonstrated biological activity in pharmacokinetic and therapeutic evaluation models, supporting the influence of genotype on chayote phytochemical composition.	[41]
Fruit tissues (mainly parenchymatous pulp region)	Isolation/characterization of a natural hydrocolloid fraction (mucilage/pectin-rich matrix)	Natural aqueous mucilage release and histochemical characterization	Fresh fruits were sectioned and evaluated for structural distribution of mucilage, pectin, starch and inulin using specific staining approaches (cresyl blue for mucilage; ruthenium red for pectates).	NR	Mucilage, pectins, inulin and starch-rich polysaccharides located in fruit tissues; Demonstrated that *S. edule* fruit contains abundant hydrocolloid components with potential applications in functional foods, edible films and biomaterials.	[71]
Pulp and whole fruit	Hydroalcoholic extraction of freeze-dried samples	Methanol	Three varieties (spiny green, spineless green, yellow) were extracted and evaluated for nutritional and phytochemical characteristics.	NR	Spiny green pulp showed the highest phenolic (90.80 mg GAE/g extract) and flavonoid (13.73 mg EC/g extract) contents; extracts showed antioxidant and α-amylase inhibitory activity.	[72]
Whole fruit (including exocarp, mesocarp and seeds)	Ethanolic extraction followed by liquid–liquid partitioning and silica-gel chromatographic fractionation	95% ethanol extraction; ether partitioning; petroleum ether/chloroform and chloroform/methanol gradient purification	Fresh fruits (1 kg scale) were cut, extracted in 95% ethanol for 72 h, filtered, concentrated by rotary evaporation, fractionated with diethyl ether, and purified through silica-gel column chromatography. HPLC quantified cucurbitacins.	NR	Cucurbitacins I, B, E, and D were targeted and identified as characteristic triterpenoid compounds. This study established a standardized extraction and analytical approach for cucurbitacin profiling and demonstrated strong variation among chayote varieties, supporting genotype-based selection of bioactive-rich materials.	[73]
Fruit mucilage (surface exudate)	Direct mucilage collection	No extraction solvent	Fresh chayote fruits were cut to stimulate mucilage exudation. The released mucilage was collected, purified, and incorporated into hydrogel/adhesive film formulations.	NR	Natural polysaccharides forming mucilage matrix; Mucilage demonstrated film-forming, adhesive, and hydrogel-forming potential, indicating applications in biomedical biomaterials.	[74]
Seed	Conventional solvent extraction (maceration; tincture, fluid extract, alcoholature, and decoction)	Ethanol (70–96%), hydroalcoholic mixtures, and water (decoction)	Fresh and dried seeds extracted according to the Farmacopea Argentina procedures; extracts evaluated for antioxidant and antiradical activities	NR	Seed aqueous extracts showed strong antioxidant activity. β-carotene bleaching inhibition reached approximately 90%, while strong hydrogen-donating activity was observed in the DPPH assay (IC_50_ ≈ 2 μg/mL).	[48]
Stems	Conventional solvent extraction (maceration)	Water, methanol, ethyl acetate, and hexane	Fresh shoots extracted separately with each solvent; extracts evaluated for phytochemicals, antioxidant activity, and α-amylase inhibition	Water extract showed the highest extraction yield	Hexane extract showed the strongest antioxidant activity. Methanol and ethyl acetate extracts exhibited the highest α-amylase inhibitory activity (IC_50_ values: 0.62 mg/mL and 2.50 mg/mL, respectively). Cooking treatments, especially steaming, improved retention of bioactive compounds.	[75]
Fruit (whole fruit extract)	Conventional solvent extraction	Methanol extract (hydroalcoholic phenolic extract)	Fresh fruit samples were homogenized and extracted with methanol. The crude extract was concentrated and subjected to phytochemical analysis using HPLC.	NR	The extract showed significant DPPH radical scavenging activity and antioxidant protection of a phospholipid membrane model against hypochlorous acid-induced oxidation. It also demonstrated anti-inflammatory potential by inhibiting oxidative damage pathways.	[11]
Seed	Bioactivity-guided extraction followed by acetone fractionation, gel filtration, affinity chromatography, and RP-HPLC	Acetone followed by chromatographic purification buffers	Sequential purification using acetone fractionation, gel filtration, affinity chromatography, and reverse-phase HPLC	46% recovery of trypsin inhibitory activity; 0.05% protein yield	Purified low-molecular-weight trypsin inhibitors (SETI-IIa, SETI-IIb, and SETI-V) exhibited potent trypsin inhibitory activity with dissociation constants of 5.4 × 10^−11^ M and 1.1 × 10^−9^ M, demonstrating their potential as bioactive protease inhibitors for nutraceutical and pharmaceutical applications.	[49]

Abbreviations: ABTS, 2,2′-azino-bis(3-ethylbenzothiazoline-6-sulfonic acid); CE, catechin equivalents; CUPRAC, cupric ion reducing antioxidant capacity; DEAE-52, diethylaminoethyl cellulose-52; DPPH, 2,2-diphenyl-1-picrylhydrazyl; DW, dry weight basis; EC_50_, half-maximal effective concentration; ESI, electrospray ionization; FRAP, ferric reducing antioxidant power; GAE, gallic acid equivalents; HPLC, high-performance liquid chromatography; IC_50_, half-maximal inhibitory concentration; MS, mass spectrometry; NR, not reported; TLC, thin-layer chromatography; TOF, time-of-flight; UAE, ultrasound-assisted extraction.

**Table 3:** Targeted mechanisms and potential health benefits of *S. edule* in different experimental models.

Disease/Condition	Experimental Model	Dose/IC_50_	Major Targeted Mechanisms	Key Biological Effects	References
Cervical cancer	HeLa human cervical cancer cells (in vitro); normal human lymphocytes	Methanolic fruit extract: 1.25–5.00 µg/mL; IC_50_ = 1.85 µg/mL (HeLa); IC_50_ = 30.04 µg/mL (lymphocytes)	↑ Cucurbitacin D-enriched fractions, ↑ antiproliferative activity, ↑ selective cytotoxicity, ↓ HeLa cell viability	↓ Cancer cell proliferation, ↑ growth inhibition (>80%), ↑ selectivity toward cancer cells, ↓ toxicity to normal lymphocytes	[43]
Breast cancer	MCF-7 human breast cancer cells (in vitro)	Hexane, dichloromethane, and methanolic fruit extracts; IC_50_ (DCM extract) = 0.58 µg/mL; significant inhibition: DCM ≥ 0.07 µg/mL, hexane ≥ 0.60 µg/mL, methanol ≥ 1.20 µg/mL	↑ Cucurbitacins (I, B, D, E), ↑ flavonoids, ↑ tannins, ↑ bioactivity-guided fractionation, ↓ MCF-7 cell viability, ↑ active fractions (F3 and F5)	↓ Cancer cell proliferation, ↑ antiproliferative activity, ↑ potency of DCM extract, ↑ efficacy of purified fractions, ↓ cell growth comparable to doxorubicin	[70]
Breast cancer	MCF-7 and MDA-MB-231 human breast cancer cells (in vitro)	Methanolic fruit extract and chromatographic fractions; concentrations evaluated according to fraction activity; IC_50_ values varied among fractions, with cucurbitacin-rich fractions showing the greatest potency	↑ Cucurbitacins B, D, E, and I, ↑ flavonoids, ↑ selective cytotoxicity, ↑ active fraction potency, ↓ cancer cell viability, ↓ proliferation	↓ MCF-7 and MDA-MB-231 cell growth, ↑ antiproliferative activity, ↑ efficacy of bioactive fractions, ↑ potential against hormone-dependent and triple-negative breast cancer cells	[41]
Cancer (preclinical safety evaluation)	CD-1 mice (in vitro/in vivo toxicity models)	Methanolic fruit extract: 300–5000 mg/kg (mice); various concentrations for *A. salina* and *D. melanogaster* assays	↑ Cucurbitacins (B, D, E, I), ↑ flavonoids, ↑ safety profile, ↓ acute toxicity, ↓ genotoxicity	High safety margin, ↓ mortality, ↓ toxic manifestations, ↑ suitability for further anticancer development	[44]
Leukemia, fibrosarcoma, and cervical cancer	P388 (murine leukemia), L-929 (murine fibrosarcoma), HeLa (human cervical cancer) (in vitro)	Crude fruit extracts of eight *S. edule* varietal groups; dose-dependent evaluation (IC_50_ not reported)	↑ Antiproliferative activity, ↑ varietal-dependent cytotoxicity, ↑ cucurbitacin-rich genotypes, ↓ tumor cell viability	↓ Proliferation of P388, L-929 and HeLa cells; ↑ identification of highly bioactive varietal groups (e.g., *nigrum spinosum*, *amarus silvestrys*); ↑ selection of promising germplasm for anticancer research	[43]
Leukemia	P388, J774, and WEHI-3 leukemia cell lines (in vitro); normal bone marrow mononuclear cells (BM-MNCs)	IC_50_ < 1.3 µg/mL (all leukemia cell lines); no significant effect on BM-MNCs	↑ Apoptotic body formation, ↑ phosphatidylserine externalization, ↑ DNA fragmentation, ↑ selective apoptosis, ↓ leukemia cell viability	↓ Proliferation of P388, J774 and WEHI-3 cells, ↑ cancer-cell selectivity, ↓ toxicity to normal BM-MNCs, ↑ potential antileukemic activity	[93]
Cervical cancer and fibrosarcoma	HeLa and L-929 cells (in vitro)	0.047, 0.23, 0.47, 1.18 and 2.37 mg/mL	↑ Bioactivity-guided fractionation, ↑ antiproliferative activity, ↑ cytotoxic fractions, ↓ cancer cell viability, ↑ fatty acid ester-enriched fraction	↓ HeLa and L-929 cell proliferation, ↑ dose-dependent cytotoxicity, ↑ identification of active antiproliferative fractions	[96]
Hypertension	Spontaneously hypertensive rats (in vivo)	Fresh *S. edule* fruit; dietary administration (dose reported in original paper)	↓ Blood pressure, ↑ vasorelaxation, ↑ diuresis	↓ Systolic blood pressure, ↑ antihypertensive activity	[97]
Endothelial dysfunction/hypertension	Angiotensin II-induced endothelial dysfunction in mice	Acetone root fraction	↓ Oxidative stress, ↓ endothelial dysfunction, ↑ NO bioavailability, ↓ vascular inflammation	↑ Endothelial function, ↓ vascular damage	[91]
Hepatic injury/NASH	Angiotensin II-induced liver injury in C57BL/6J mice	11 mg/kg/day hydroalcoholic root extract	↓ TNF-α, ↓ IL-1β, ↓ IL-6, ↓ TGF-β, ↓ fibrosis, ↓ hepatic TG accumulation	↑ Hepatoprotection, ↓ steatosis, ↓ inflammation, ↓ fibrosis	[67]
Hepatic steatosis and insulin resistance	Oleic acid-treated HepG2 cells	Hydroalcoholic root extract	↑ AMPK activation, ↓ p-IRS(Ser312), ↑ lipolysis, ↓ triglyceride accumulation, ↓ oxidative stress	↑ Insulin sensitivity, ↓ steatosis, ↓ inflammatory response	[68]
Diabetes mellitus	STZ-induced diabetic mice	*Sechium* hybrid extract	↓ Hyperglycemia, ↑ antioxidant defense, ↓ lipid peroxidation, ↑ nephroprotection, ↑ cardioprotection	↓ Blood glucose, ↑ lipid metabolism, ↑ hepato-, nephro- and cardioprotection	[86]
Pancreatic dysfunction/diabetes	STZ-induced diabetic mice and MIN-6 β-cells	Chayote juice	↓ ROS, ↓ β-cell apoptosis, ↑ insulin secretion, ↑ antioxidant enzymes	↑ Pancreatic protection, ↑ glucose homeostasis	[98]
Hepatic lipid metabolism/metabolic syndrome	Oleic acid-treated HepG2 cells	Polyphenol extract from shoots	↑ AMPK activation, ↓ SREBP-1c, ↓ FAS, ↑ lipolysis, ↓ lipogenesis	↓ Hepatic lipid accumulation, ↑ lipid metabolism	[82]
Diabetic nephropathy and nephrotoxicity	STZ-induced diabetic mice; gentamicin- and potassium dichromate-induced nephrotoxicity	Aqueous leaf extract (200 mg/kg)	↓ Serum creatinine, ↓ BUN, ↓ uric acid, ↑ renal antioxidant defense	↑ Nephroprotection, ↑ renal histology, ↓ diabetic renal damage	[99]

Abbreviations: Ang II, angiotensin II; AMPK, AMP-activated protein kinase; *A. salina*, *Artemia salina*; BM-MNCs, bone marrow mononuclear cells; BUN, blood urea nitrogen; C57BL/6J, C57 black 6J mouse strain; DCM, dichloromethane; *D. melanogaster*, *Drosophila melanogaster*; FAS, fatty acid synthase; HepG2, human hepatocellular carcinoma cell line; IC_50_, half-maximal inhibitory concentration; IL, interleukin; MCF-7, Michigan Cancer Foundation-7 human breast adenocarcinoma cell line; MDA-MB-231, M. D. Anderson-Metastatic Breast-231 human triple-negative breast cancer cell line; MIN-6, mouse pancreatic β-cell line; mmHg, millimeters of mercury; NASH, nonalcoholic steatohepatitis; NO, nitric oxide; p-IRS (Ser312), phosphorylated insulin receptor substrate at serine 312; P388, murine lymphocytic leukemia cell line; ROS, reactive oxygen species; SREBP-1c, sterol regulatory element-binding protein 1c; STZ, streptozotocin; TG, triglycerides; TGF-β, transforming growth factor beta; TNF-α, tumor necrosis factor-α; WEHI-3, murine myelomonocytic leukemia cell line. ↑ upregulation; ↓ downregulation.

## Data Availability

No new data were created or analyzed in this study. Data sharing is not applicable to this article.

## Abbreviations

The following abbreviations are used in this manuscript:

8-OHdG	8-Hydroxy-2′-deoxyguanosine
ABA	Abscisic acid
ABTS	2,2′-Azino-bis(3-ethylbenzothiazoline-6-sulfonic acid)
ADME	Absorption, distribution, metabolism, and excretion
AKT	Protein kinase B
AMPK	AMP-activated protein kinase
AUC	Area under the curve
BM-MNCs	Bone marrow mononuclear cells
CAT	Catalase
CP	Chayote pectin
CSPI	Chayote seed protein isolate
CTS	Chayote tuber starch
DM	Diabetes mellitus
DPPH	2,2-Diphenyl-1-picrylhydrazyl
DW	Dry weight
EG	Experimental group
FoxO1	Forkhead transcription factor O1
FRAP	Ferric reducing antioxidant power
G6Pase	Glucose-6-phosphatase
GAE	Gallic acid equivalents
GLP-1	Glucagon-like peptide-1
GPx	Glutathione peroxidase
GPR43	G-protein-coupled receptor 43
HbA1c	Glycated hemoglobin
HDL-C	High-density lipoprotein cholesterol
HOMA-IR	Homeostasis model assessment of insulin resistance
LAB	Lactic acid bacteria
LD50	Lethal dose 50
LXRα	Liver X receptor alpha
MetS	Metabolic syndrome
MMP-9	Matrix metallopeptidase 9
Nrf2	Nuclear factor erythroid 2-related factor 2
NUS	Neglected and Underutilized Species
OSI	Oxidative stress index
OxS	Oxidative stress
PEPCK	Phosphoenolpyruvate carboxykinase
PG	Placebo group
PI3K	Phosphatidylinositol 3-kinase
RCT	Randomized controlled trial
ROS	Reactive oxygen species
RSM	Response surface methodology
SCFAs	Short-chain fatty acids
SIRT	Sirtuin
SOD	Superoxide dismutase
SSR	Simple sequence repeat
TAS	Total antioxidant status
TBARS	Thiobarbituric acid reactive substances
TFC	Total flavonoid content
T2DM	Type 2 diabetes mellitus
TOS	Total oxidant status
ToLCNDV	Tomato leaf curl New Delhi virus
TPC	Total phenolic content
UAE	Ultrasound-assisted extraction
WGD	Whole-genome duplication
WVP	Water vapor permeability
